# Construction of C-C bonds via photoreductive coupling of ketones and aldehydes in the metal-organic-framework MFM-300(Cr)

**DOI:** 10.1038/s41467-021-23302-w

**Published:** 2021-06-11

**Authors:** Tian Luo, Lili Li, Yinlin Chen, Jie An, Chengcheng Liu, Zheng Yan, Joseph H. Carter, Xue Han, Alena M. Sheveleva, Floriana Tuna, Eric J. L. McInnes, Chiu C. Tang, Martin Schröder, Sihai Yang

**Affiliations:** 1 Department of Chemistry, University of Manchester, Manchester, UK; 2 Department of Nutrition and Health, China Agricultural University, Beijing, China; 3 Institute of Molecular Sciences and Engineering, Institute of Frontier and Interdisciplinary Science, Shandong University, Qingdao, China; 4 College of Biological, Chemical Sciences and Engineering, Jiaxing University, Jiaxing, China; 5 Diamond Light Source, Harwell Science Campus, Oxfordshire, UK; 6 Photon Science Institute, University of Manchester, Manchester, UK

**Keywords:** Heterogeneous catalysis, Photocatalysis

## Abstract

Construction of C-C bonds via reductive coupling of aldehydes and ketones is hindered by the highly negative reduction potential of these carbonyl substrates, particularly ketones, and this renders the formation of ketyl radicals extremely endergonic. Here, we report the efficient activation of carbonyl compounds by the formation of specific host-guest interactions in a hydroxyl-decorated porous photocatalyst. MFM-300(Cr) exhibits a band gap of 1.75 eV and shows excellent catalytic activity and stability towards the photoreductive coupling of 30 different aldehydes and ketones to the corresponding 1,2-diols at room temperature. Synchrotron X-ray diffraction and electron paramagnetic resonance spectroscopy confirm the generation of ketyl radicals via confinement within MFM-300(Cr). This protocol removes simultaneously the need for a precious metal-based photocatalyst or for amine-based sacrificial agents for the photochemical synthesis.

## Abstract (web summary)

The construction of C-C bonds via reductive coupling carbonyl compounds is a huge challenge in organic transformations. Here, the authors develop a highly efficient system for the photoreductive coupling of aldehydes and ketones to the corresponding 1,2-diols under mild conditions.

## Introduction

**Fig. 1:**
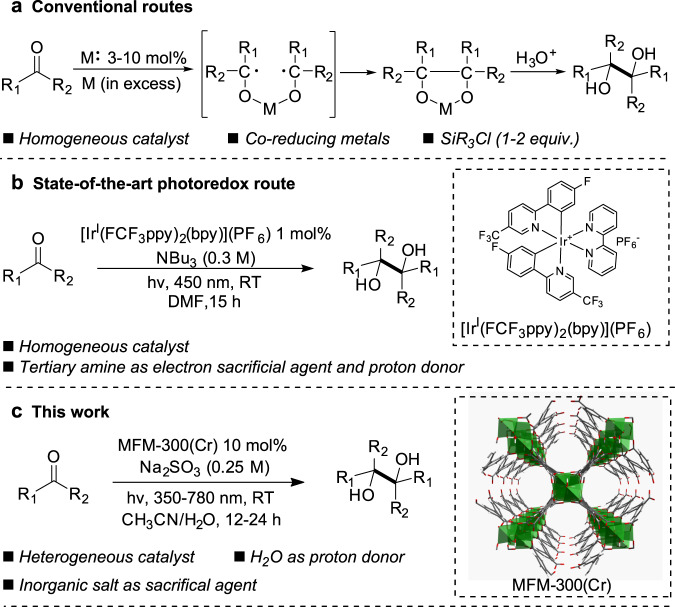
Comparison of methods for the reductive coupling of aldehydes and ketones. **a** Conventional routes involving the use of a metal salt as catalyst (M: = MgCl_2_, SmI_2_, NiCl_2_) and more than stoichiometric amounts of co-reducing metals (M = Mg, Al, Mn, Sm). 1–2 equivalents of SiR_3_Cl is also required to prevent catalyst inhibition through the coordination of diol^60^. **b** Current state-of-the-art photoreductive route catalysed by organometallic complexes based upon Ir(I) and Ru(II) in homogenous systems^9^. **c** Heterogeneous photoreductive route catalysed by MFM-300(Cr) described herein.

The construction of C–C bonds is of paramount importance in organic chemistry, and photochemistry can enable efficient chemical conversion and synthesis using solar energy^1,2^. Aldehydes and ketones are among the most valuable organic compounds that can be obtained from renewable resources, such as biomass, in large scale. Reductive coupling of aldehydes and ketones offers a promising route to the synthesis of 1,2-diols, which are important structural motifs in a broad range of natural products^3^, pharmaceuticals^4^, protease inhibitor^5^, peptidomimetics^6^ and polymers^7^, and are widely used as chiral ligands and auxiliaries in organic reactions^8^. However, this is a very challenging photochemical process owing to the highly negative reduction potential of carbonyl compounds, particularly for ketones [e.g., acetophenone: *E*_1/2red_ = −1.79 V vs. normalised hydrogen electrode (NHE)^9^] and to date, success has only been achieved in exceptional cases^9–14^. Various photocatalysts have been reported including poly(*p*-phenylene)^12^, [Cu^I^(pipes)(BINAP)]BF_4_ [pyzs = 5-(4-fluorosulfonyl)amino-3-(2-pyridyl)-pyrazole; BINAP = (rac)-2,2′-bis(diphenylphosphino)-1,1′-binaphthalene]^13^ and Pt(II)-based complexes^14^. The most efficient photocatalyst [Ir^I^(FCF_3_ppy)_2_(bpy)](PF_6_) [ppy = 2-(2-pyridinyl-N)phenyl-C, bpy = 2,2NHE-bipyridyl]^9^ promotes the reductive coupling of aldehydes and ketones in dimethylformamide (DMF) by using tributylamine (NBu_3_) as the sacrificial electron donor under visible light (450 nm) irradiation (Fig. 1). However, such homogenous catalysts incorporate expensive precious metals, and often suffer from low solubility and limited stability upon contact with moisture and/or air^15^.

Metal-organic framework (MOF) materials that adopt stable three-dimensional (3D) open-framework structures and semiconductor-like behaviour have been exploited as emerging photocatalysts^16^. Upon light irradiation, the organic ligands of MOFs can serve as antennas to harvest light, generate photo-induced electrons and activate the metal centre, thus facilitating the “ligand-to-metal charge-transfer” (LMCT)^17^. More importantly, the micropore environment offers a unique platform to activate the substrates via confined adsorption arising  from the specific host–guest interactions^18–20^. The impact of host–guest interactions on the activation of adsorbed substrates and catalytic activity in supramolecular cages^21–23^ and zeolites^24,25^ have been widely studied. In contrast, the impact on confined catalysis of host–guest structures in MOFs is less well defined^26,27^. The design features of MOFs affords tremendous potential as photocatalysts for a range of redox reactions such as CO_2_ conversion^28^, hydrogen/oxygen evolution^29^, degradation of organic molecules^30^, benzene hydroxylation of benzene^31^, oxidation of benzylamine and alcohols^32^ and selective hydrogenation of olefins^33^. However, only a few successes have been achieved in the construction of C–C bonds through photoreactions using MOF catalysts.

Here, we report the efficient photoreductive coupling of a broad range of aldehydes and ketones (30 substrates) over a hydroxyl-decorated MOF, MFM-300(Cr), in H_2_O/CH_3_CN using an inorganic salt, sodium sulphite (Na_2_SO_3_), as the reductant at room temperature. The confined adsorption and activation of carbonyl substrates via the formation of strong host–guest hydrogen bonds promote their conversion into ketyl radicals within the MOF pores, which has been studied by synchrotron X-ray diffraction and electron paramagnetic resonance (EPR) spectroscopy, affording key insights into the mechanism of catalysis. MFM-300(Cr) shows excellent optical photoelectrical properties, enabling the use of a broad spectrum of light (350–780 nm) to drive the photoredox process. Tertiary amine-based sacrificial agents^34^ are not required in the above catalytic reactions and this greatly simplifies the post-reaction isolation of products and gives a reduced environmental impact on photosynthesis.

## Results

### Synthesis and characterisation of catalysts

MFM-300(Cr), [Cr_2_(OH)_2_(L)] (H_4_L = biphenyl-3,3′,5,5′-tetracarboxylic acid), was synthesised by hydrothermal reaction of CrCl_3_·6H_2_O and H_4_L in acidic (HCl) water at 210 °C^35^. The purity of bulk material has been confirmed by powder X-ray diffraction (PXRD) and thermogravimetric analysis (TGA) (Supplementary Figs. 1 and 2). MFM-300(Cr) comprises of chains of corner-sharing [CrO_4_(OH)_2_] octahedral linked by *cis*-µ_2_-OH groups, and these chains are further bridged by tetracarboxylate ligands to form a ‘wine rack’ array. The pores have a diameter of ~7.5 Å taking into account van der Waals radii (Supplementary Fig. 3). Scanning electron microscopy (SEM) shows cube-shaped crystals (0.5–2 μm) for as-synthesised MFM-300(Cr) samples (Supplementary Fig. 4), while desolvated MFM-300(Cr) exhibits a surface area of 1045 m^2^ g^−1^ based upon N_2_ sorption isotherm data (Supplementary Fig. 5).

**Fig. 2:**
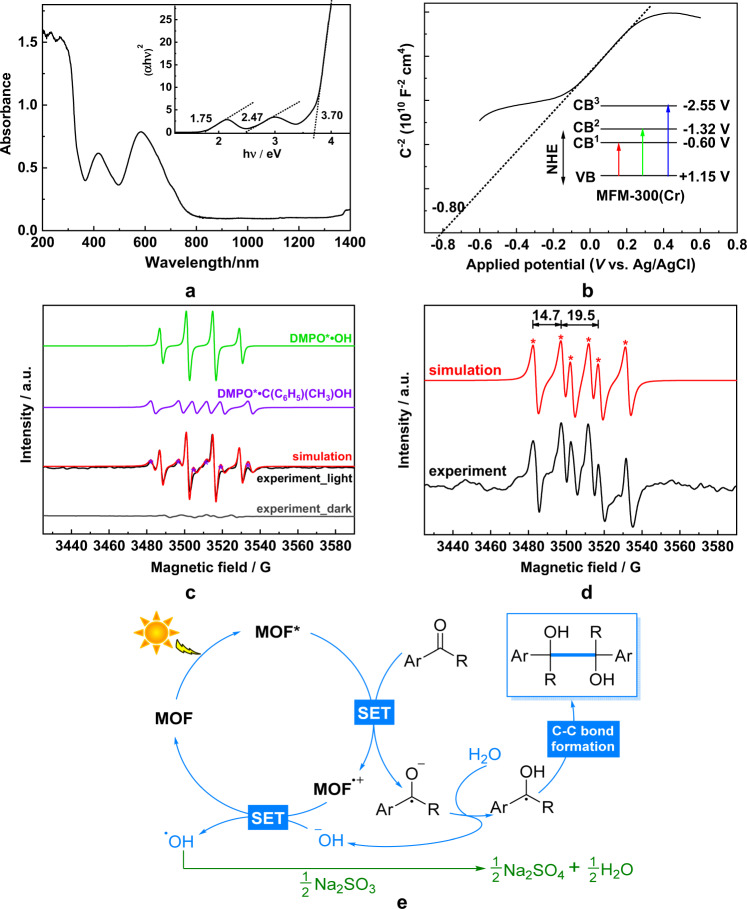
Physical characterisation and mechanism study. **a** The UV-Vis diffuse reflectance spectrum of MFM-300(Cr): inset figure shows a plot for the bandgap calculation. **b** Mott–Schottky plot of MFM-300(Cr) at frequency of 1 kHz. **c** X-band EPR spectra of in situ photocatalytic reaction with 13% H_2_O: experimental data before and after irradiation (black), simulated spectrum (red) comprising two components: DMPO*•C(C_6_H_5_)(CH_3_)OH (simulation, purple) and DMPO*•OH (simulation, green). **d** X-band EPR spectrum of in situ photocatalytic reaction without H_2_O, highlighting hyperfine coupling to ^14^N and ^β^H. The latter is characteristic of an adduct with a C-centred radical. **e** Proposed mechanism of photocatalytic synthesis of diols over MFM-300(Cr). SET = single electron transfer.

MFM-300(Cr) was chosen as a photocatalyst because of its high stability and optical properties among the iso-structural family of MFM-300(M^III^) (M = Al, Cr, V, Fe, Ga, In)^36–39^ materials. It is the only one that retains high crystallinity and stability after irradiation for 2 days under the photochemical conditions used in this work (Supplementary Fig. 6). Additionally, solid-state UV-Vis diffuse reflectance spectroscopy data confirm that MFM-300(Cr) has the widest window of light absorption up to 800 nm (Fig. 2a). The UV-Vis diffuse reflectance spectra of CrCl_3_·6H_2_O and H_4_L have also been measured (Supplementary Fig. 7). Three absorption bands centred at 289, 453 and 624 nm are observed for CrCl_3_·6H_2_O, which can be assigned to the *d–d* transitions of Cr^3+^, i.e., ^4^A_2g_ $\to$ ^4^T_2g_, ^4^A_2g_ $\to$ ^4^T_1g_ (*F*) and ^4^A_2g_ $\to$ ^4^T_2g_ (*P*)^40^, respectively. The absorption at 310 nm is assigned to π → π* transition of the phenyl rings of the ligand. The overall absorption within the ultraviolet region of the UV-Vis spectrum of MFM-300(Cr) is therefore attributed to the combination of π → π* transition of the phenyl rings of the ligand, LMCT transitions^41^ and a *d*–*d* transition of the Cr(III) centre. The two strong absorption bands in the visible light region centred at 420 and 580 nm are assigned to two additional *d*–*d* transitions of the Cr(III) centre. Compared with the precursors, the shifts of these bands (*Δ* = 33–44 nm) observed in the MOF is likely due to the hybridisation between the frontier orbitals of Cr^3+^ and the ligands. Photocatalysts with small band gaps can be readily excited by visible light (*λ* < 713 nm) to produce electron/hole pairs, thus simulating conventional semiconductor materials^42^. Importantly, MFM-300(Cr) exhibits an optical band gap (*E*_g_) of 1.75 eV (Fig. 2a and Supplementary Fig. 8), lower than that of the Al, Fe, Ga, In-analogues of MFM-300 (3.81, 2.67, 3.30 and 3.04 eV, respectively). Although adopting a similar band gap of 1.72 eV, MFM-300(V) shows poor photochemical stability and PXRD data confirmed structural degradation of the recycled sample after the photoreaction (Supplementary Fig. 6e), which is attributed to its intrinsic redox-activity^43^. The band gap for MFM-300(Cr) is higher than that of Fe-porphyrin-based MOFs (1.30 eV)^44^ but compares favourably with most MOFs and semiconductors^45^ such as chemically modified n-TiO_2_ (2.32 eV)^46^, Ag_3_PO_4_ (2.43 eV)^47^, NH_2_-MIL-125(Ti) (2.60 eV)^48^, and NH_2_-UiO-66(Zr) (2.75 eV)^49^. Overall, MFM-300(Cr) demonstrates the promising potential to promote photoreactions and we thus targeted the construction of C–C bonds by reductive coupling of aldehydes and ketones to produce 1,2-diols.

### Catalytic studies

**Fig. 3:**
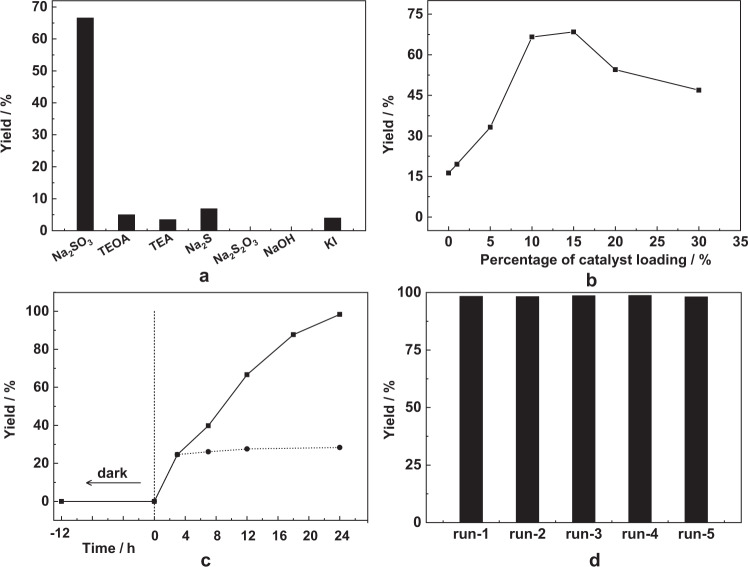
Optimisation of photocatalytic reductive coupling of acetophenone. The effects of **a** reducing agent, **b** catalyst loading, **c** reaction time including removal of MOF at 3 h; **d** recycling test and yield of product on each cycle. Reaction conditions for a typical experiment: substrate (0.50 mmol), MFM-300(Cr) (10 mol%, 0.05 mmol), CH_3_CN/H_2_O (15 mL/15 mL), Na_2_SO_3_ (0.25 M), 25 °C, 350–780 nm, light irradiation for 12 h (**a** and **b**) and for 24 h (**d**). The amount of other salts in **a** TEOA (3.0 mL, 22.6 mmol), TEA (3.0 mL, 21.5 mmol), Na_2_S or Na_2_S_2_O_3_ (7.50 mmol), NaOH (0.075 mmol, pH = 11.7).

The effects of different reducing agent, catalyst loading, reaction time and solvent on the efficiency of the photocatalytic reaction were studied using acetophenone as the model substrate owing to its highly negative reduction potential (*E*_1/2red_ = −1.79 V vs. NHE)^9^ (Supplementary Fig. 9). Tertiary amine-based organic compounds, such as triethanolamine (TEOA) and triethylamine (TEA), are widely used as sacrificial electron donors to promote photoredox reactions^9,14^. However, these organic materials are prone to oxidation to various by-products and can have significant environmental impacts when used on a large scale. The inorganic salt Na_2_SO_3_ has been used previously as a reductant in C–H/C–D exchange reactions^50,51^ but not widely for other photoredox reactions. We have tested a variety of reductants (TEA, TEOA, Na_2_SO_3_, Na_2_S, Na_2_S_2_O_3_ and KI) and found that Na_2_SO_3_ shows the best performance (yield of 67% at 12 h), which is notably better than TEOA (5%), TEA (3.5%), Na_2_S (7%) and KI (4%) under the same conditions (Fig. 3a). PXRD analysis of the recovered salt from the aqueous phase shows a mixture of Na_2_SO_3_ and a minor amount of Na_2_SO_4_ (Supplementary Fig. 10). To rule out the influence of an alkaline environment, NaOH was tested and yielded no diol product. The optimal catalyst loading and reaction time have been assessed (Fig. 3b, c), and the yield of diol initially increased with increasing catalyst loading and reached a maximum at ~10–15 mol%. Further increase in catalyst loading leads to a decrease in yield because excess MOF particles in the suspension reduce photon utilisation. The yield also increased from 67% to 98% when the reaction time was increased from 12h to 24 h with a 10 mol% catalyst loading. A biphasic system comprising of H_2_O and an organic solvent is required to dissolve Na_2_SO_3_ and the organic substrate, respectively. A series of organic solvents, including acetonitrile (CH_3_CN), tetrahydrofuran (THF), methanol (CH_3_OH), DMF, dimethylacetamide (DMA) and dichloromethane (DCM) were screened, with CH_3_CN/H_2_O affording the highest yield (Supplementary Fig. 11). We thus arrived at optimised reaction conditions with a catalyst loading of 10 mol%, Na_2_SO_3_ (0.25 M) as reducing agent, CH_3_CN/H_2_O as a solvent, and a reaction time of 12–24 h.

Control experiments were conducted at 12 h to gain additional insights (Table 1). Without Na_2_SO_3_, the substrate remains intact upon irradiation with or without MFM-300(Cr) (Entries 2, 3). This indicates that without Na_2_SO_3_ as the hole-scavenger, the photogenerated electrons and holes re-combine rapidly before transfer to the reactant. As expected, light also plays a key role and no product was obtained under dark conditions (Entries 5, 6). Low yields of the product were observed in the absence of MFM-300(Cr) (16%) or when using a powdered mixture of CrCl_3_·6H_2_O and H_4_L as photocatalyst (16%) (Entries 4, 7), suggesting that the presence of the framework structure of MFM-300(Cr) is crucial. This agrees well with the leaching test, where negligible further conversion was observed after removal of the MOF catalyst after reaction for 3h (Fig. 3c).

**Table 1:** Summary of catalysis results for control experiments.

Entry	Catalyst	Reducing agent	Wavelength (nm)	Product yield (%)	Residual substrate (%)
1	MFM-300(Cr)	Na_2_SO_3_	350–780	67	31
2	n.a.	n.a.	350–780	n.a.	98
3	MFM-300(Cr)	n.a.	350–780	n.a.	97
4	n.a.	Na_2_SO_3_	350–780	16	83
5	n.a.	Na_2_SO_3_	Dark	n.a.	98
6	MFM-300(Cr)	Na_2_SO_3_	Dark	n.a.	98
7	CrCl_3_·6H_2_O-H_4_L	Na_2_SO_3_	350–780	16	72

Reaction conditions: acetophenone (0.50 mmol), MFM-300(Cr) (10 mol%, 0.05 mmol), CH_3_CN/H_2_O (15 mL/15 mL), Na_2_SO_3_ (0.25 M), 25 °C, 350-780 nm, 12 h. For entry 7, a powdered mixture of CrCl_3_·6H_2_O (0.1 mmol, 0.027 g) and H_4_L (0.05 mmol, 0.0165 g) were used (H_4_L = biphenyl-3,3,5,5-tetracarboxylic acid).

**Fig. 4:**
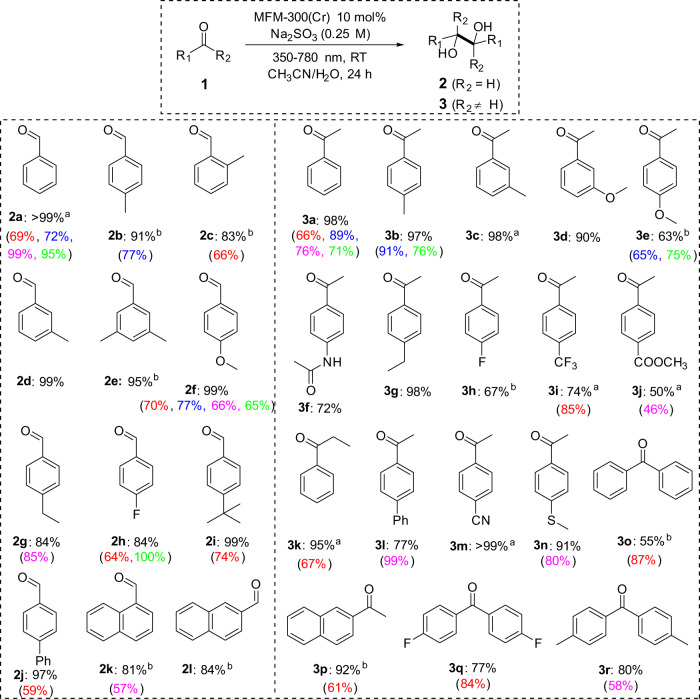
Yields for photoreductive coupling of aldehydes and ketones over MFM-300(Cr). The yields of corresponding diols are listed under the chemical structure for each substrate, and these were determined by NMR spectroscopic analysis of the reaction mixture using an internal standard (details are given in the Supplementary Information). The data for reaction conversion and yields of by-product (if any) are also given in the Supplementary Information. Reaction conditions: substrate (0.50 mmol), MFM-300(Cr) (10%, 0.05 mmol), CH_3_CN/H_2_O (15 mL/15 mL), Na_2_SO_3_ (0.25 M), 25 °C, 350–780 nm. The reaction time is 24 h unless noted as ^a^36-48 h or ^b^12 h. For comparison, the yields of the same reaction from experiments reported in the literature are given below in brackets. In red^9^: [Ir^I^(FCF_3_ppy)_2_(bpy)](PF_6_) (1 mol%), NBu_3_ (3 equiv), DMF, 450 nm, RT, 15 h; in blue^14^: Pt(II) complex (1 mol%), DIPEA (diisopropylethylamine, 2 equiv), DMF, >370 nm, 6 h; in purple^13^: Cu(pypzs)(BINAP)BF_4_ (2 mol%), Hantzsch ester (2 equiv.), THF, blue LED, 18 h; in green^60^: (t-BuO)_3_FeK (0.05 eq), Mg, SiR_3_Cl (1.5 eq), THF, RT, 12 h.

The performance of this photochemical system has been studied using 30 different substrates (Fig. 4), and significantly, a broad range of aldehydes and ketones with electron-donating or electron-withdrawing substituents afford yields of diol product. The substitution of -H with -methyl group at *para-*, *meta-*, and *ortho-* positions did not show an apparent impact in either aldehydes or ketones, except for 2-methylacetophenone which gives no product due to likely steric hindrance effects. Interestingly, various polybenzene substrates based upon naphthalene (2k, 2l and 3p) and biphenyl (3o, 3q and 3r) show excellent yields of diols over a relatively short reaction time (12 h). It should be noted that a range of factors can jointly influence the overall catalytic efficiency and selectivity, including the stability of intermediates, reductive potentials, steric effects and electrophilic properties of the substrate. The main by-products from these reactions are the corresponding mono-alcohols of reduced substrates, and the ratios of *meso*/*dl* isomers are given in the Supplementary Information. Overall, these results are notably superior to those afforded using precious-metal-based complexes under homogeneous conditions^9,14^ (Fig. 4).

### Determination of binding domains for adsorbed carbonyl substrates

**Fig. 5:**
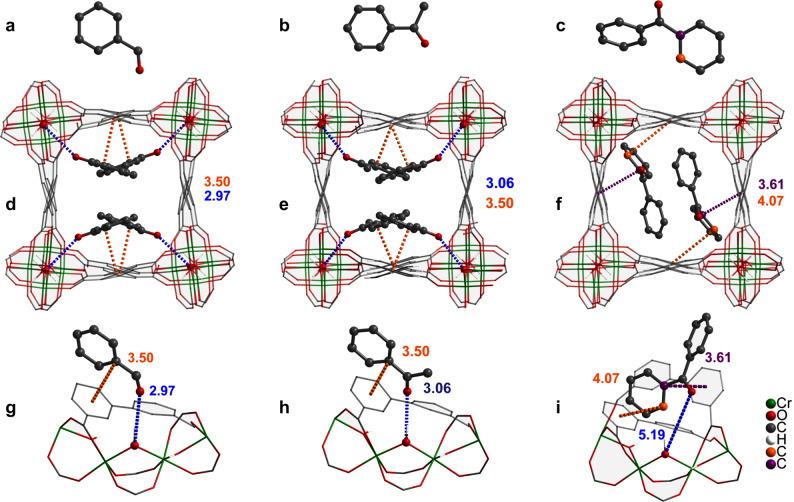
Views of crystal structures of substrate-loaded MFM-300(Cr). All models were obtained from Rietveld refinements based on SPXRD data. Hydrogen atoms are omitted for clarity. Distances for hydrogen bonding are given as the donor-to-acceptor distances, given the uncertainty in the position of protons. **a**–**c** The chemical structures of PhCHO, PhCOCH_3_ and Ph_2_CO, respectively. **d**–**f** View of the structures of MFM-300(Cr)·1.56PhCHO, MFM-300(Cr)·1.56PhCOCH_3_ and MFM-300(Cr)·0.81 Ph_2_CO. **g**–**i** Detailed views of host–guest binding of adsorbed PhCHO, PhCOCH_3_ and Ph_2_CO, respectively, within MFM-300(Cr). The minor amounts of free water molecules in **d**, **e**, **g**, **h** are omitted for clarity. The guest molecules are highlighted by using an amplified ball-and-stick model. Two carbon atoms in Ph_2_CO are highlighted in purple and orange to illustrate the host–guest interaction.

To investigate the location of adsorbed substrates within the pores of MFM-300(Cr), synchrotron powder X-ray diffraction (SPXRD) was undertaken on four samples of MFM-300(Cr) loaded with H_2_O–CH_3_CN (v/v = 50/50), acetophenone (PhCOCH_3_), benzaldehyde (PhCHO) and benzophenone (Ph_2_CO). Full structural analyses of all SPXRD data have yielded highly satisfactory Rietveld refinements (Supplementary Figs. 12–14, Supplementary Tables 1–5). All guest-loaded MFM-300(Cr) samples show full retention of the framework structure (Fig. 5). Solvent molecules (H_2_O–CH_3_CN) show a disordered distribution in the pores of MFM-300(Cr) and are primarily stabilised by host–guest [H_2_O···O(H)–Cr = 2.88(1) Å] and guest–guest hydrogen bonds [H_2_O···NCCH_3_ = 2.45(7) Å] (Supplementary Fig. 15). This suggests that Na_2_SO_3_ can potentially access the hydrophilic channels of MFM-300(Cr) in the presence of H_2_O to accelerate charge-transfer. Adsorbed benzaldehyde and acetophenone molecules interact primarily with the bridging –OH groups of MFM-300(Cr) through their carbonyl groups [C=O···O(H)–Cr = 2.97(3), 3.06(5) Å, respectively] to form hydrogen bonds in PhCHO@MFM-300(Cr) and PhCOCH_3_@MFM-300(Cr). These adsorbed molecules are further stabilised by parallel-displaced π···π interactions between the benzene rings of guest molecules and ligands of MFM-300(Cr) with an inter-planar distance of 3.50(2) and 3.50(3) Å for adsorbed benzaldehyde and acetophenone, respectively. Also, trace amounts of free water molecules were found in the structures of PhCHO@MFM-300(Cr) and PhCOCH_3_@MFM-300(Cr), interacting with adsorbed carbonyl substrates via hydrogen bonding. In Ph_2_CO@MFM-300(Cr), adsorbed Ph_2_CO molecules are stabilised by the electrostatic interaction between the benzene ring of the guest molecule and the two adjacent ligands with an inter-planar distance of 3.61(1) and 4.07(2) Å, respectively. There is a hydrogen bond interaction between the carbonyl oxygen of Ph_2_CO and bridging –OH groups of MFM-300(Cr) [C=O···O(H)–Cr = 5.19(1) Å]. Thus, the SPXRD study confirms significant host–guest-binding interactions between the heavily confined substrate molecules and MFM-300(Cr), which have been further confirmed by FTIR (Supplementary Fig. 16 and see the “Discussion” section). The strong host–guest interaction would accelerate the rate of single electron transfer (SET), and thus promote the photocatalytic efficiency.

### Photoelectrical characterisations of MFM-300(Cr)

Mott–Schottky (MS) analysis^52^ was conducted to determine the position of the conduction band (CB) of MFM-300(Cr) (Fig. 2b and Supplementary Fig. 17). The positive slope of the MS plot indicates that MFM-300(Cr) is an *n*-type semiconductor and the extrapolation of the MS plot yielded a flat-band potential of −0.60 V vs. NHE, close to the bottom of CB (*E*_CB_). Based on the bandgap of 1.75 eV, the position of the valence band (*E*_VB_) of MFM-300(Cr) is estimated to be at +1.15 V vs. NHE (Fig. 2b), which is in excellent agreement with that (+1.1 V vs. NHE) as measured by valence-band X-ray photoelectron spectroscopy (VB-XPS) (Supplementary Fig. 18). Based on the additional two absorption bands observed in Fig. 2a, another two excited states could be generated with excitation energies of −1.32 and −2.55 V vs. NHE^53^. Upon light irradiation at 350–780 nm, the MOF is activated and electrons in the VB are promoted to the CB, creating holes at the VB. These holes at an oxidation potential of +1.15 V vs. NHE are highly oxidising and can be readily recharged by sacrificial electron donors. The photo-induced electrons with three reductive potentials of −0.60, −1.32 and −2.55 V vs. NHE can reduce carbonyl substrates with *E*_1/2red_ = −1.42, −1.79 and −1.63 V vs. NHE for benzaldehyde, acetophenone and Ph_2_CO^9^, respectively. Importantly, the confinement and binding of these substrates within MFM-300(Cr) result in their efficient activation and rapid reduction into ketyl radicals (see below). The close contact between the host framework and guest substrate can also facilitate the transfer of the photo-induced electrons, and thus prevent electron–hole recombination, thereby maximising catalytic efficiency. Interestingly, it has been recently reported that twisted amides exhibit significantly enhanced reactivity inside confined molecular cages owing to the strong host–guest interactions^21^. Therefore, the judicious use of host–guest interactions in confined porous catalysts can effectively tune the reactivity of substrates.

### EPR spectroscopy

To investigate the mechanism of reaction, in situ EPR spin trapping was used to detect the radicals generated under photocatalytic conditions. 5,5-Dimethyl-1-pyrroline-n-oxide (DMPO) was used as the spin trap: this is a nitrone which reacts with short-lived radicals to generate stable nitroxyl free radicals^54^. The EPR spectra of the generated DMPO-adducts are dominated by hyperfine coupling to the ^14^N of the nitroxyl group and to the β-hydrogen. The β-position is also the site of the trapping, and the ^β^H coupling is very sensitive to the nature of the trapped radical. Using acetophenone as the substrate under optimised photocatalytic conditions as discussed above, a significant amount of •OH radicals was detected (Supplementary Fig. 19 and Supplementary Table 6; DMPO–OH has similar ^14^N and ^β^H couplings). The high concentration of •OH radical is derived from water/hydroxides oxidised by photogenerated holes^55^, which results in two beneficial effects: (i) diminished hole–electron recombination due to the rapid consumption of photogenerated holes, and therefore (ii) enhanced generation of photo-induced electrons. In order to test whether the efficient trapping of •OH radicals was hiding the formation of other radical species, similar experiments were performed with a decreased water content of 13%. A second species (Fig. 2c) was observed with a much larger ^β^H coupling, which is characteristic of a trapped carbon-centred radical and is assigned to DMPO*•C(C_6_H_5_)(CH_3_)OH (Supplementary Fig. 20). The EPR signal centred at *g* = 2.0055 is in excellent agreement with the reported hyperfine constant parameters^56^ (Supplementary Table 6). This species is found cleanly in an experiment in the absence of water (Fig. 2d). No radical was captured for reactions conducted under dark conditions. Thus, a carbon-centred ketyl radical has been identified as the reaction intermediate to construct C–C bonds in 1,2-diols within MFM-300(Cr).

## Discussion

The structural diversity and flexibility in the design of metal–ligand coordination in MOFs create an excellent route to explore new multifunctional photocatalysts that integrate the semiconductor-type photoelectrical properties with functional pore environments. A highly efficient system to promote the construction of C–C bonds to afford the corresponding diols through photoreductive coupling of a large family of aldehydes and ketones over MFM-300(Cr) under mild conditions (25 °C, 350–780 nm light) has been achieved. The use of precious metal-based photocatalysts and organic amine-based sacrificial agents in organic media has been replaced by a recyclable MOF, a simple inorganic salt, and an equimolar mixture of H_2_O/CH_3_CN. The introduction of H_2_O into the photochemical system has the added advantage of acting as hydrogen donors to the product. MFM-300(Cr) shows excellent catalytic stability over five cycles, with a negligible decrease in the product yield or crystallinity of recycled MFM-300(Cr) observed (Fig. 3d and Supplementary Fig. 6a). A combination of diffuse reflectance and EPR spectroscopy, SPXRD and MS have confirmed that the carbonyl substrates can be preferentially adsorbed within the pore of MFM-300(Cr), facilitating efficient substrate activation and desirable catalyst–substrate charge-transfer to produce ketyl radicals. Furthermore, spatial confinement within MFM-300(Cr) is beneficial for the coupling of ketyl radicals to give final products in excellent yields.

## Methods

### Synthesis of MFM-300(Cr)-solvate, [Cr_2_(OH)_2_(C_16_O_8_H_6_)](H_2_O)_6_

CrCl_3_·6H_2_O (200 mg, 0.751 mmol) and H_4_L (70.0 mg, 0.212 mmol) were added to a mixture of H_2_O (10 mL) and HCl (1%, 1.5 mL) and the suspension stirred at room temperature for 30 min before being transferred into a 23 mL Teflon autoclave and heated at 210 °C for 3 days. After cooling to room temperature, the blue microcrystalline solid was collected via filtration, briefly washed with H_2_O (20 mL × 2) and acetone (20 mL × 2), and dried in air (yield: 87%). Elemental analysis (% cal./found): [Cr_2_(OH)_2_(C_16_H_6_O_8_)](H_2_O)_6_ (Cr 18.2/20.3, C 33.6/33.6, H 3.52/3.90, N 0.0/0.0). Selected ATR-IR (Supplementary Fig. 21): ν/cm^−1^: 3583(w), 3078(w), 1700(m), 1633(m), 1591(m), 1531(s), 1424(vs), 1373(vs), 1324(w), 1265(m), 1161(w), 1087(w), 914(w), 769(m), 728(m), 718(m), 663(m).

### Determination of optical band gaps

The UV–Vis diffuse reflectance spectroscopic data were recorded on a UV–Vis spectrophotometer (Shimadzu, UV 2600) equipped with an integrating sphere using BaSO_4_ as reference. The optical band gaps were calculated under the hypothesis of Kubelka–Munk equation (1) ^57^ and Tauc plot (2):

1 $${F}({{R}}_{\infty })={(1-{{R}}_{\infty })}^{2}/2{{R}}_{\infty }$$

2 $${[{F}({{R}}_{\infty }){h}\nu ]}^{2}={B}({h}\nu -{{E}}_{{\rm{g}}})$$

3 $${A}={K}\cdot {F}({{R}}_{\infty })$$

where *R*_∞_, *F*(*R*_∞_), *hν*, *B* and *E*_g_ refer to the reflectance of the sample, the Kubelka–Munk function, the photon energy, a constant and the optical band gap, respectively. The relationship between the absorption (*A*) and *F*(*R*_∞_) is expressed using Eq. (3), where *K* is a constant. Absorption (*A*) is directly obtained from UV-Vis diffuse reflectance spectroscopy. The Tauc plot gives the value of band gap (*E*_g_) by extending the tangent of the (*αhν*)^2^ curve and intersecting with the *hν* axis. The diffuse reflectance spectroscopic measurements were conducted on three samples of MFM-300(Cr) prepared from different batches. This gave consistent results for the band gap analysis (Supplementary Fig. 22).

### Electrochemical studies

Electrochemical measurements were performed on a CHI660E electrochemical workstation. A three-electrode cell system was employed, including a pre-prepared glassy carbon working electrode, a Pt-electrode counter electrode and a Ag/AgCl reference electrode. In a typical process for preparing the working electrode, 4.0 mg of MFM-300(Cr) was dispersed in a solution of 970 μL ethanol and 30 μL Nafion (5 wt%). After being ultrasonically treated for 30 min, 30 μL of the resultant slurry was dropped on the surface of the glass carbon. After drying in air for 12 h, the working electrode was used to perform the electrochemical measurements in 0.5 M Na_2_SO_4_ electrolyte solution. MS analysis was conducted at different frequencies of 1k, 2k, 3k. 4k, and 5k Hz.

### Photocatalytic reaction

A 300 W Xe lamp (Zhongjiaojinyuan Co., Ltd) was used at a wavelength of 350–780 nm, and the distance between the lamp and quartz round bottom flask was fixed to 5.0 cm. A broad range of aldehydes and ketones with electron-donating or electron-withdrawing substituents (Supplementary Fig. 23) were tested. In a typical photocatalytic reaction, Na_2_SO_3_ (0.25 M) and a fixed amount of desolvated MFM-300(Cr) powder were dispersed in a quartz round bottom flask (50 mL) filled with a solution of CH_3_CN/H_2_O (15 mL/15 mL) and sonicated for 5 min. The substrate (0.5 mmol) was added to the above suspension, sealed and heated to 25° Cusing a water bath. The lamp (300 W, *λ* = 350–780 nm) was turned on to trigger the photoreaction as a function of time. Upon completion, the MOF catalyst was recycled by centrifugal separation, washed with acetone (10 mL × 3) and then dried in an oven overnight for additional tests. The unreacted substrate and reaction product from the filtrate was extracted using DCM (15 mL × 3). The DCM-filtrate was combined and removed by rotary evaporation, and the residue dissolved in DMSO-*d*^*6*^ or CDCl_3_ and analysed by ^1^H NMR spectroscopy using nitromethane as internal standard and high-resolution mass spectrometry (HRMS). For the leaching test, the MOF catalyst was removed from the system after 3 h in a typical experiment by centrifugal separation. The filtrate was transferred back into the flask and sealed, and the photoreaction continued for an additional 21 h. The reaction was processed and products analysed using the same method as above.

### EPR spectroscopy 

Continuous-wave EPR spectroscopy was carried out at X-band (9.85 GHz) using an EMX micro-spectrometer (Bruker). EPR data were collected at room temperature with a modulation amplitude of 10 G, with a microwave  frequency of ~9.85 GHz and power of ~6.325 mW. Strong pitch (*g* = 2.0028) was used as a reference. Theoretical modelling of EPR spectra was performed using the EasySpin toolbox (Version 5.2.24) in Matlab^58^.

All reagents were deoxygenated under Ar. In a typical experiment, DMPO (176.7 mmol/L) was dissolved in CH_3_CN and used as a spin trap. Acetophenone (0.050 mmol) and Na_2_SO_3_ (0.25 M) were dissolved in a mixed solution of CH_3_CN/H_2_O (1.5 mL/1.5 mL) and MFM-300(Cr) (0.005 mmol) was added. 0.2 mL of the resultant mixture was injected into an Ar deoxygenated vial, followed by 0.1 mL of the DMPO solution. 0.1 mL of the resultant DMPO mixed solution was then transferred into a capillary for freeze pumping to further degas the solution in order to remove all the dissolved gases (<0.01 mbar). The capillary was then directly used for EPR measurements. EPR spectra of the system were collected before (dark) and after (light) radiation (350–780 nm) by a Xe lamp for 10 min.

### Structure determination and refinements of synchrotron PXRD data

Synchrotron PXRD experiments were undertaken on Beamline I11 Diamond Light Source (Oxford, UK) [*λ* = 0.826562(2) Å]. Desolvated MFM-300(Cr) was obtained by heating the solvated sample under a dynamic vacuum at 120 °C overnight. To prepare the substrate-loaded samples, the desolvated MOF was dispersed in acetophenone (liquid), benzaldehyde (liquid) or Ph_2_CO (dissolved in CH_3_CN). After being soaked for 2 days, the MOF was isolated and dried. The powder sample was loaded in a 0.7 mm borosilicate glass capillary. High-resolution synchrotron PXRD data were collected in the 2*θ* range 0–150° with 0.002° data steps using multi-analyser crystal detectors at 25.0 °C. The PXRD patterns were refined using the Rietveld method in TOPAS software. Stephen fitting^59^ was applied to describe the diffraction peaks and their anisotropic broadening. The scale factor and lattice parameters were allowed to refine for all the diffraction patterns. The refined structural parameters include the fractional coordinates (*x*, *y*, *z*), the isotropic displacement factors for all the atoms, and the site occupancy factors for the framework and guest molecules. The final stage of the Rietveld refinement involved soft restraints to the C–C bond lengths within the benzene rings, and rigid body refinement was applied to the guest molecules in the pore. The quality of the Rietveld refinements was confirmed by the low weighted profile factors and the good fit to the data with reasonable isotropic displacement factors within experimental error (Supplementary Tables 1–5).

### Reporting summary

Further information on research design is available in the Nature Research Reporting Summary linked to this article.

## Supplementary information

Supplementary Information

Reporting Summary

## Data Availability

The data that support the findings of this study are available from the corresponding authors on reasonable request.
