# Supplementary material for: Construction of C-C bonds via photoreductive coupling of ketones and aldehydes in the metal-organic-framework MFM-300(Cr)
Source: Nat Commun. 2021 Jun 11;12:3583. doi: 10.1038/s41467-021-23302-w (PMC8196067; doi:10.1038/s41467-021-23302-w)
Supplement: Supplementary file 1 — Supplementary Information [file 41467_2021_23302_MOESM1_ESM.pdf]

Supplementary Information

Construction of C-C bonds *via* photoreductive coupling of ketones and aldehydes in the metal-organic-framework material MFM-300(Cr).

Tian Luo,[1] Lili Li,[1] Yinlin Chen,[1] Jie An[2]*, Chengcheng Liu,[1,3] Zheng Yan,[1,4] Joseph H. Carter,[1,5] Xue Han[1], Alena M. Sheveleva[1,6], Floriana Tuna[1,6], Eric J. L. McInnes[1,6], Chiu C. Tang[5], Martin Schröder[1]* and Sihai Yang[1]*

1. Department of Chemistry, University of Manchester, Manchester, M13 9PL (UK)

2. Department of Nutrition and Health, China Agricultural University, Beijing, 100193 (China)

3. Institute of Molecular Sciences and Engineering, Institute of Frontier and Interdisciplinary Science, Shandong University, Qingdao, 266237 (China)

4. College of Biological, Chemical Sciences and Engineering, Jiaxing University, Jiaxing, 314001 (China)

5. Diamond Light Source, Harwell Science Campus, Oxfordshire, OX11 0DE (UK)

6. Photon Science Institute, University of Manchester, Manchester, M13 9PL (UK)

Supplementary Methods

Materials. $CrCl_3 \cdot 6H_2O$ (>98%) was purchased from Acros Organics. Hydrochloric acid (37%), acetonitrile (>99%), sodium sulphide nonahydrate ($Na_2S \cdot 9H_2O$, >98.0%), $Na_2S_2O_3$ (>98.0%), triethanolamine (TEOA, >99.0%) were purchased from Sigma-Aldrich Co., UK. N, N-dimethylformamide (DMF, >99%), sodium sulphite (Na_2SO_3, 98%), sodium hydroxide pearl (NaOH, >97%), dichloromethane (DCM), triethylamine (TEA, laboratory reagent grade) were bought from Fisher Chemical. Biphenyl-3,3',5,5'-tetracarboxylic acid (H_4L) was synthesised according to the literature procedure.[1] All chemicals and reagents used in this experiment were used as received without further purification.

Material characterisations. PXRD data were collected on an X-ray powder diffractometer (Phillips X'pert MPD) with Cu-Kα radiation at a scan speed of 1.5°/min. TGA measurements were performed under N_2 flow (5 mL/min) at a heating rate of 10 ºC/min from 25 to 600 ºC on a Pyris1 thermogravimetric analyser (Perkin-Elmer). Elemental analysis for C, H and N content of MFM-300(Cr) were carried out using a CE-440 Elemental Analyser manufactured by Exeter Analytical. ICP-OES measurements for analysis of Cr were carried out using a Perkin-Elmer Optima 2000. ^{1}H NMR and ^{13}C NMR spectra were measured on Bruker B500 or B400 spectrometers. The recycled MFM-300(Cr) sample was characterised using the same techniques as the fresh sample. FTIR spectra were recorded using a Bruker Alpha II FT-IR spectrophotometer. Samples were prepared using the same method as described for the synchrotron X-ray diffraction experiments.

Supplementary Figures

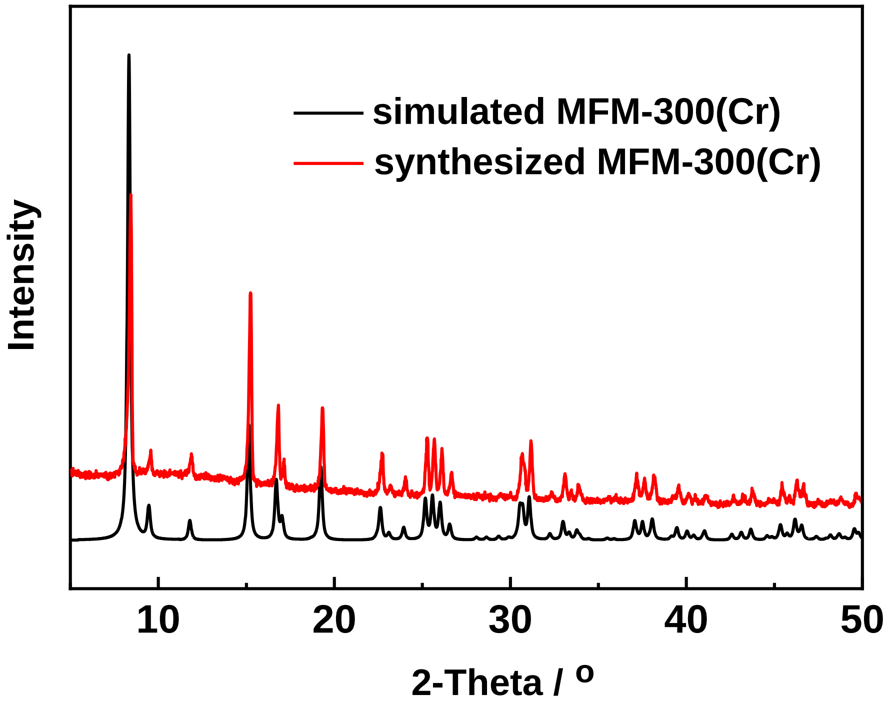

Supplementary Fig. 1. PXRD patterns of simulated and as-synthesised MFM-300(Cr).

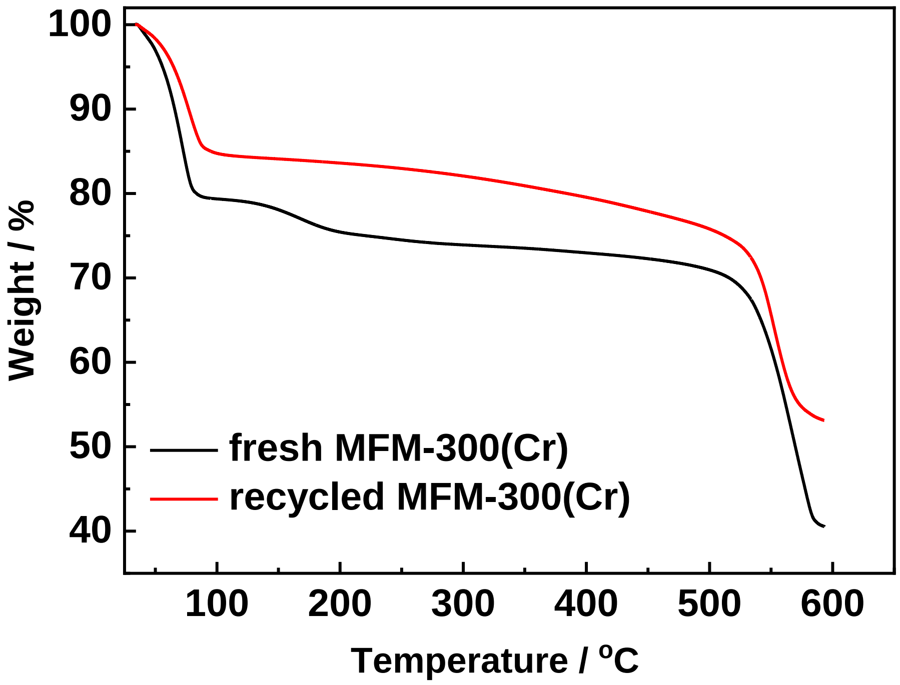

Supplementary Fig. 2. TGA plots of fresh and recycled MFM-300(Cr) samples (after one cycle of photocatalysis).

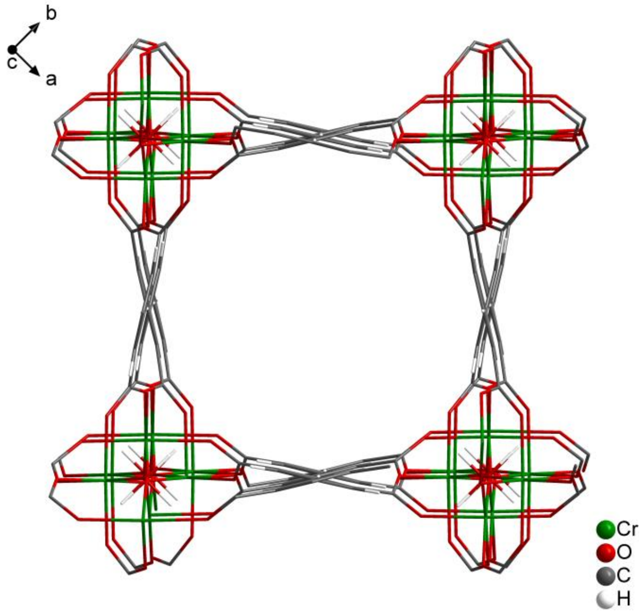

Supplementary Fig. 3. View of the crystal structure of MFM-300(Cr) along the *c* axis showing pore size of ~7.5 Å.

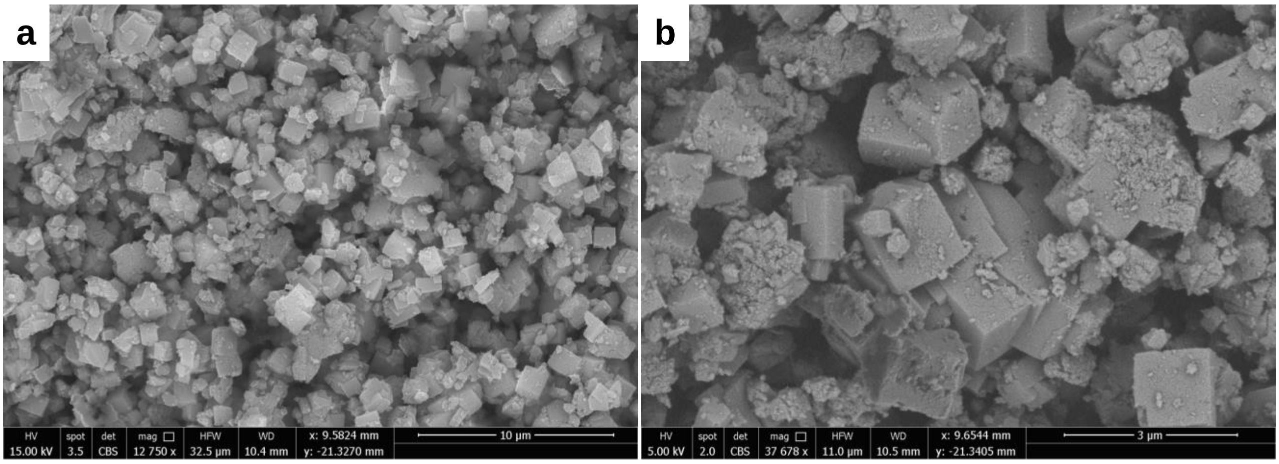

Supplementary Fig. 4. SEM images of as-synthesised MFM-300(Cr). The scale bar in a and b is 10 and 3 μm, respectively.

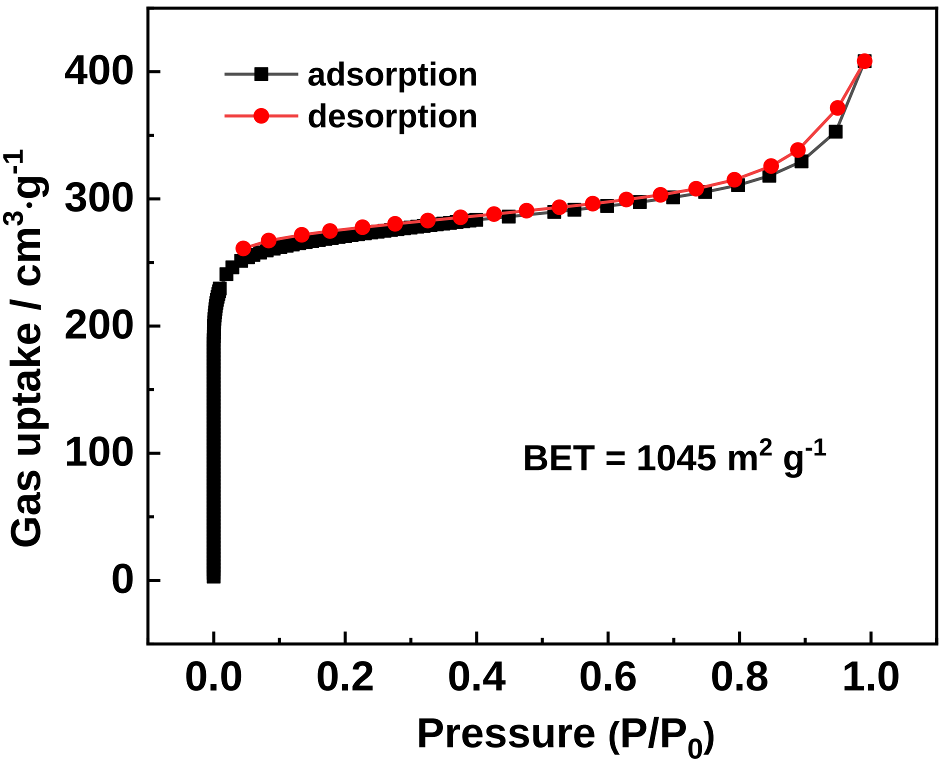

Supplementary Fig. 5. N_2 sorption isotherms of desolvated MFM-300(Cr) at 77K. The BET surface area is calculated to be 1045 m^2 g^{-1}.

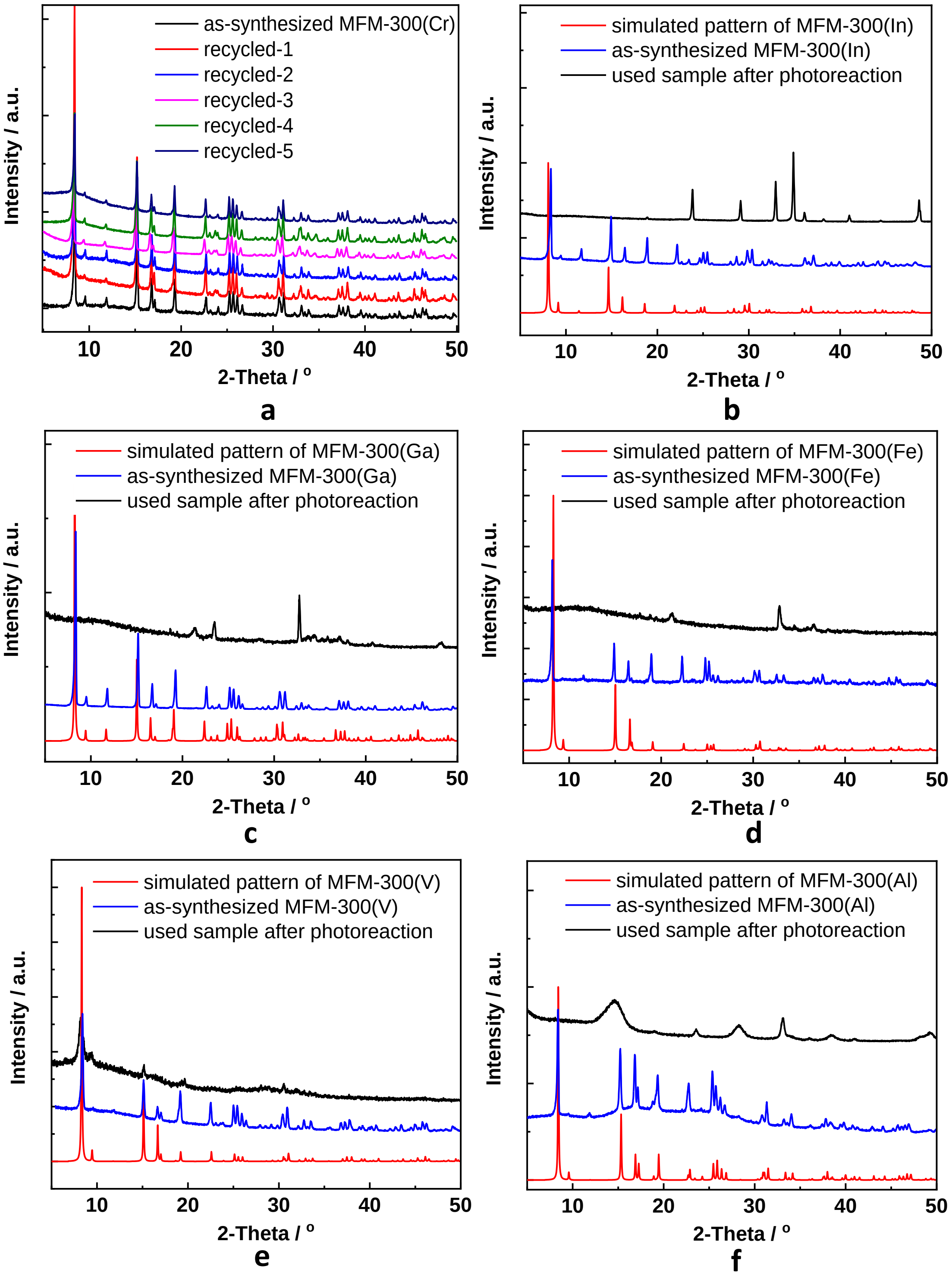

Supplementary Fig. 6. PXRD patterns of fresh and used samples of MFM-300(M) (M = Al, Ga, In, Cr, Fe, V) from recycled photocatalytic reactions using acetophenone as substrate. Reaction conditions: acetophenone (0.50 mmol), MOF (10 mol%, 0.05 mmol), CH_3CN/H_2O (15 mL/15 mL), Na_2SO_3 (0.25 M), 25 ºC, 350-780 nm, 12 h. (a) MFM-300(Cr); (b) MFM-300(In); (c) MFM-300(Ga); (d) MFM-300(Fe); (e) MFM-300(V); (f) MFM-300(Al).

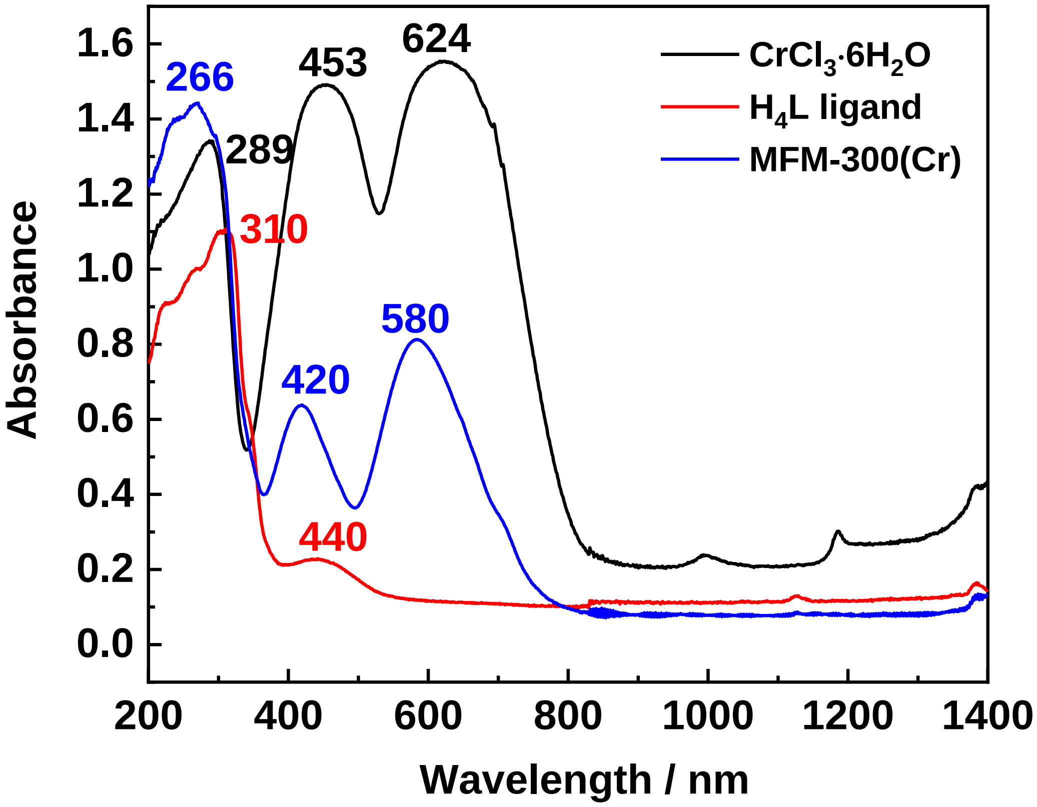

Supplementary Fig. 7. UV/Vis diffuse reflectance spectra of MFM-300(Cr), and of $CrCl_3 \cdot 6H_2O$ and H_4L used to synthesise MFM-300(Cr).

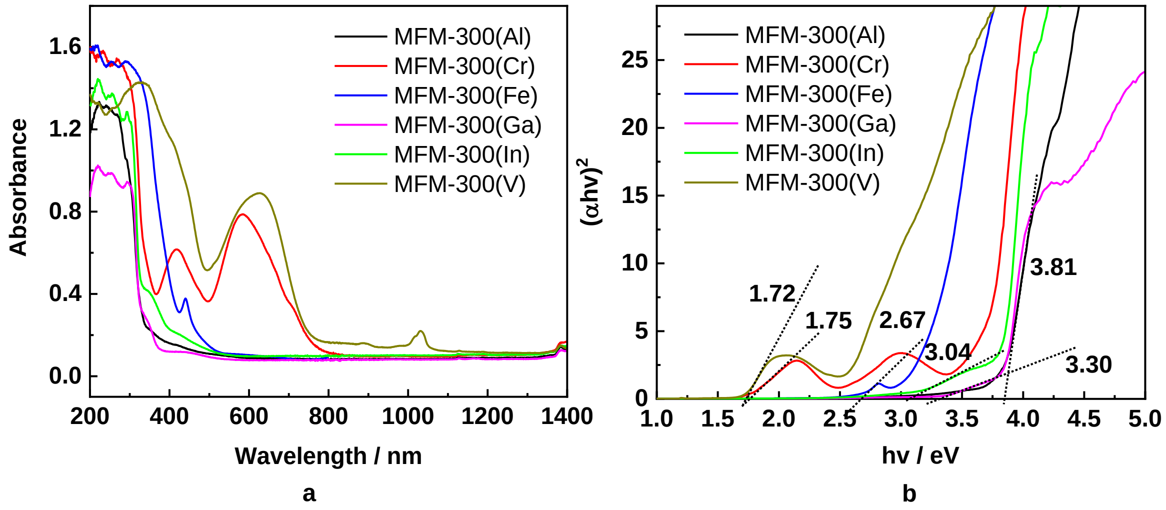

Supplementary Fig. 8. (a) The UV/Vis diffuse reflectance spectra of MFM-300(M) (M=Al, Ga, In, Cr, Fe, V); (b) the corresponding tauc plots for the calculation of band gaps.

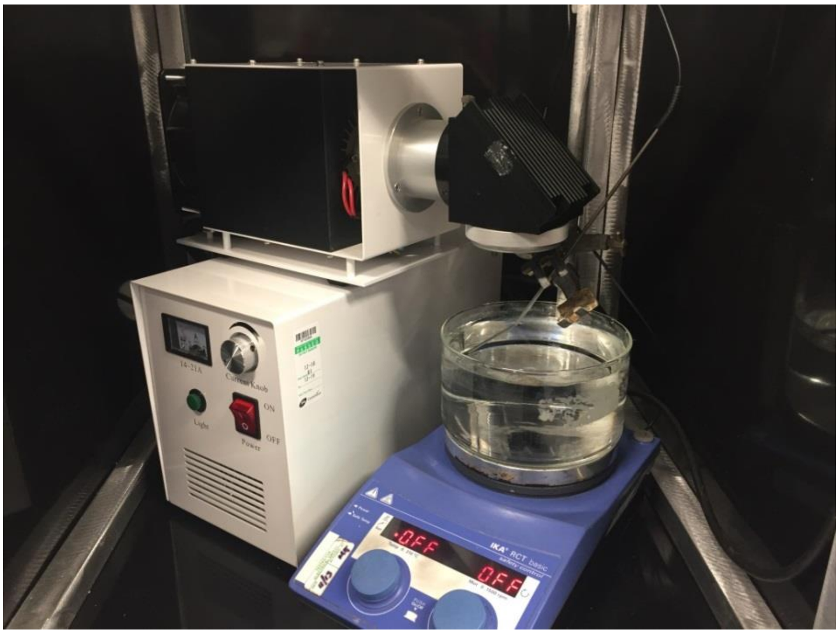

Supplementary Fig. 9. View of the set-up of photocatalytic systems used in this study. The entire system is placed in a black box.

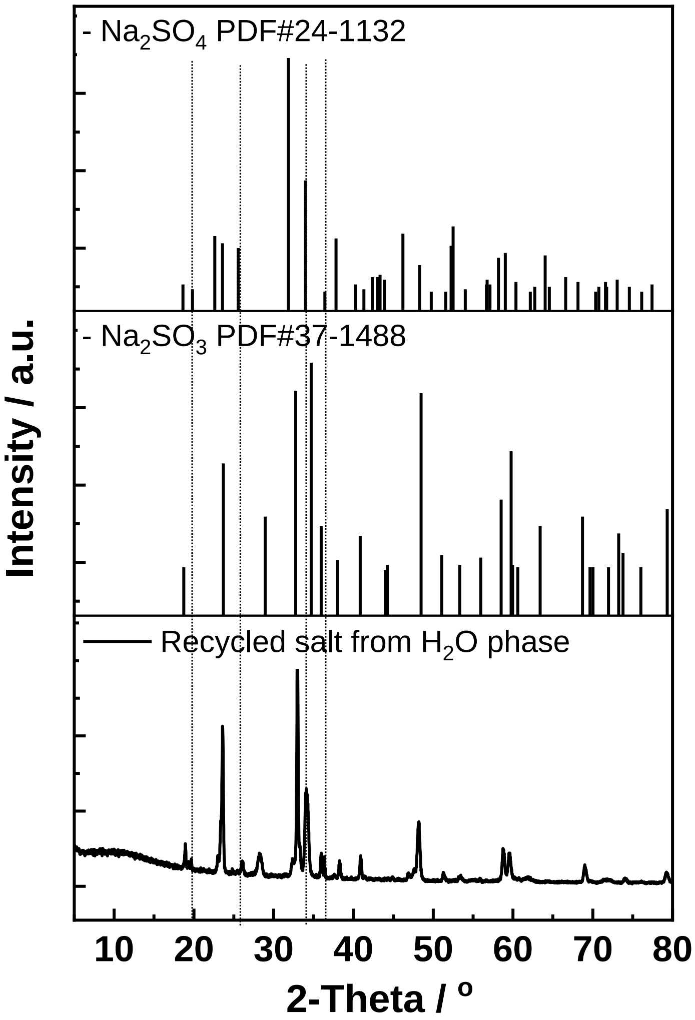

Supplementary Fig. 10. PXRD patterns of the recycled inorganic salt from the H_2O phase.

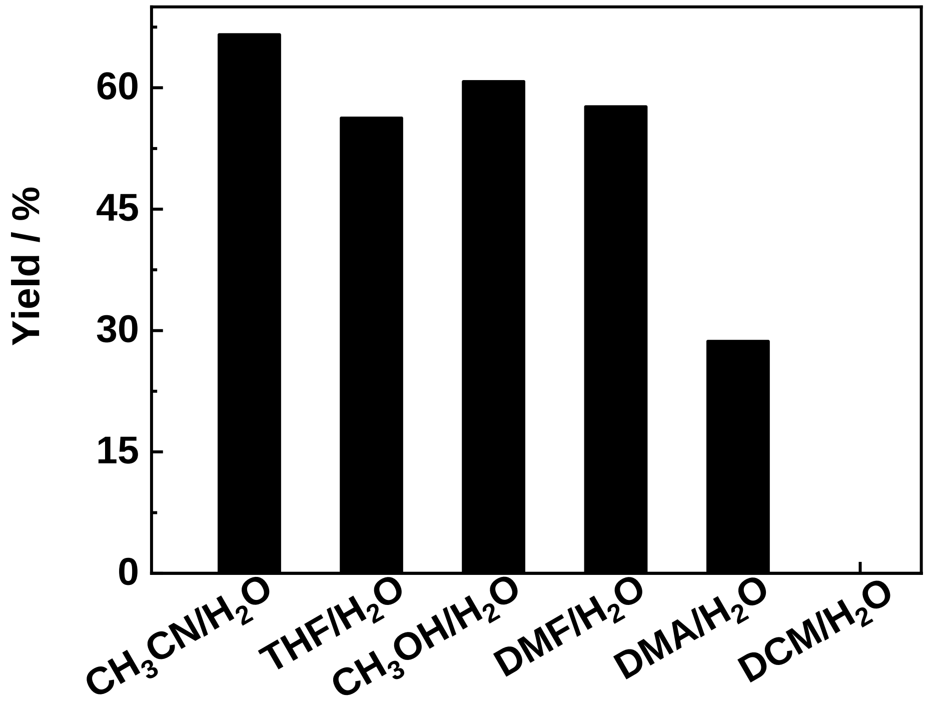

Supplementary Fig. 11. Optimisation study of the photoreductive coupling of acetophenone by using different solvent systems. Reaction conditions: acetophenone (0.50 mmol), MFM-300(Cr) (10 mol%, 0.05 mmol), CH_3CN/H_2O (15 mL/15 mL), Na_2SO_3 (0.25 M), 25 °C, 350-780 nm, 12 h.

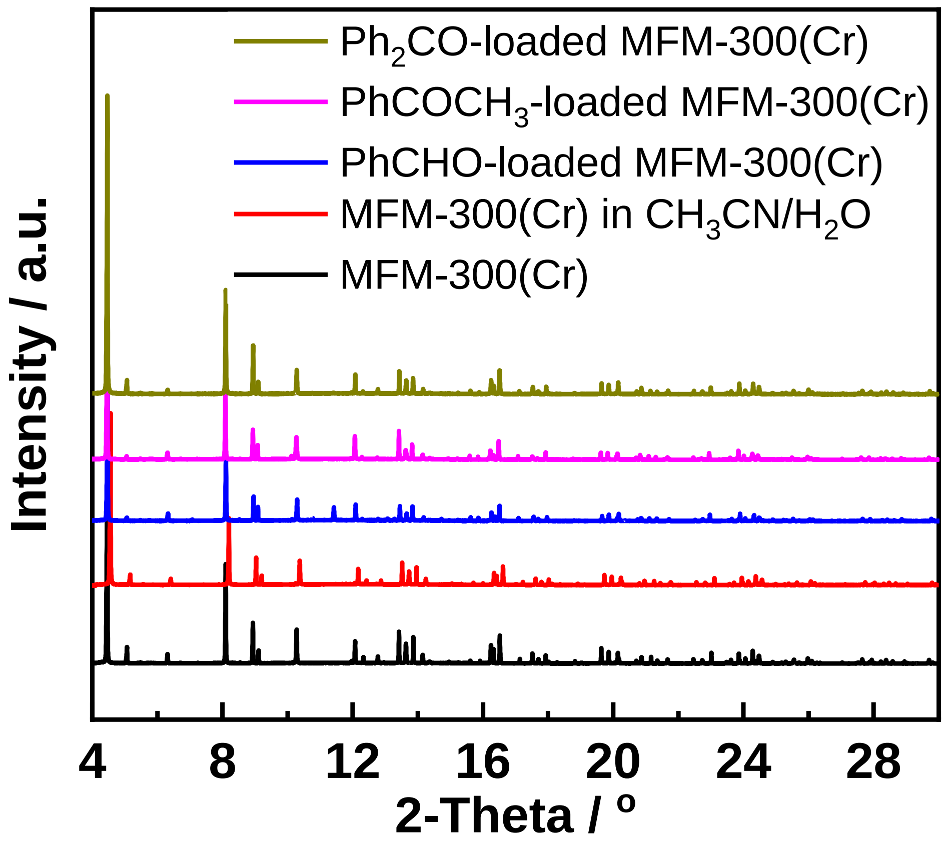

Supplementary Fig. 12. SPXRD patterns of as-synthesised and guest-loaded MFM-300(Cr) [λ = 0.826562(2) Å]. Acetophenone ($PhCOCH_3$), benzaldehyde (PhCHO) and benzophenone (Ph_2CO).

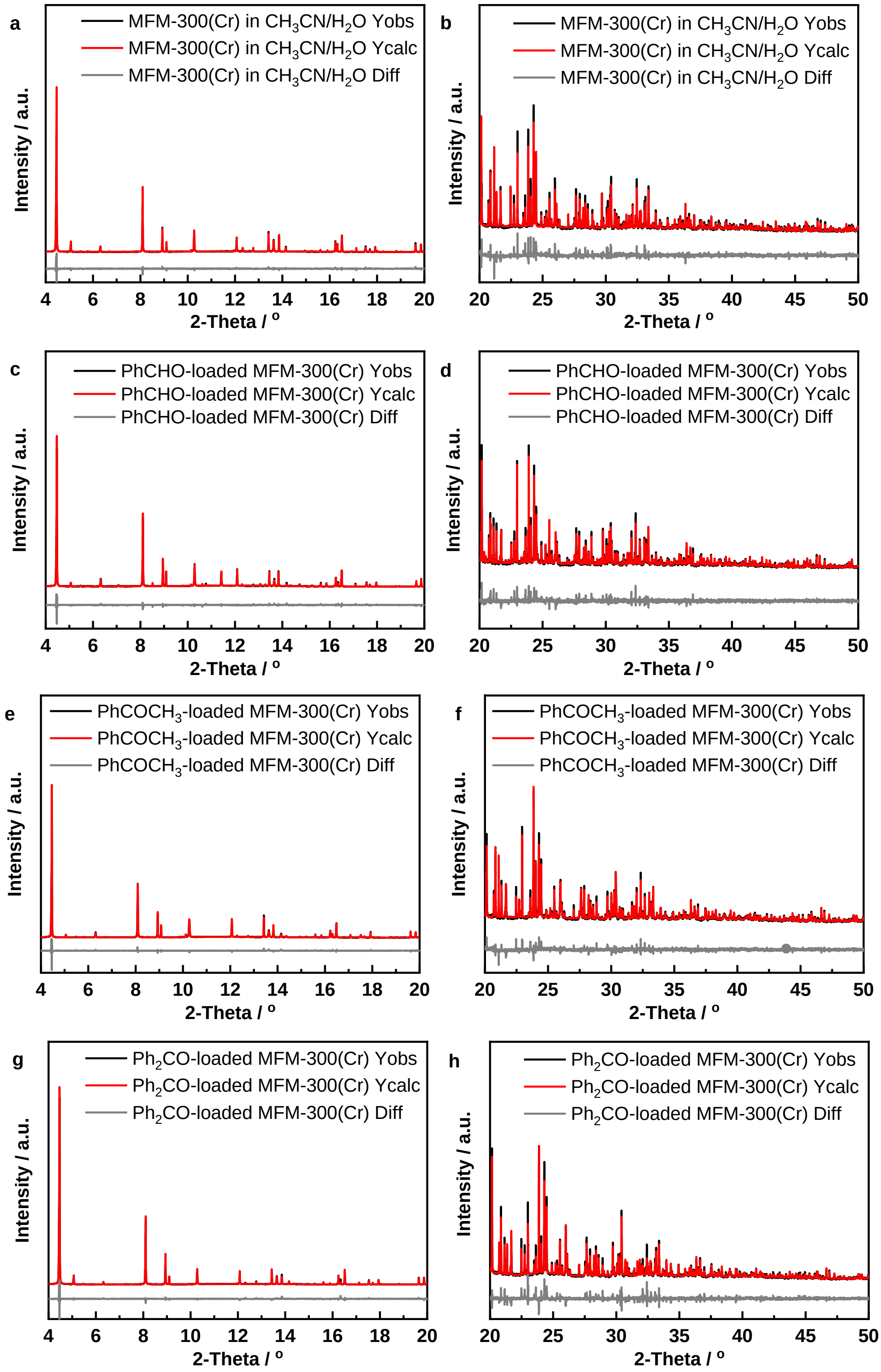

Supplementary Fig. 13. Experimental data (black line) and Rietveld refinement (red line) and the difference between them (grey line) for SPXRD patterns of (a,b) as MFM-300(Cr) in CH_3CN/H_2O, (c,d) PhCHO-loaded MFM-300(Cr), (e,f) $PhCOCH_3$-loaded MFM-300(Cr) and (g,h) Ph_2CO-loaded MFM-300(Cr) at room temperature at a 2θ range of (a,c,e,g) 3 - 20° and (b,d,f,h) 20 - 50° [λ = 0.826562(2) Å].

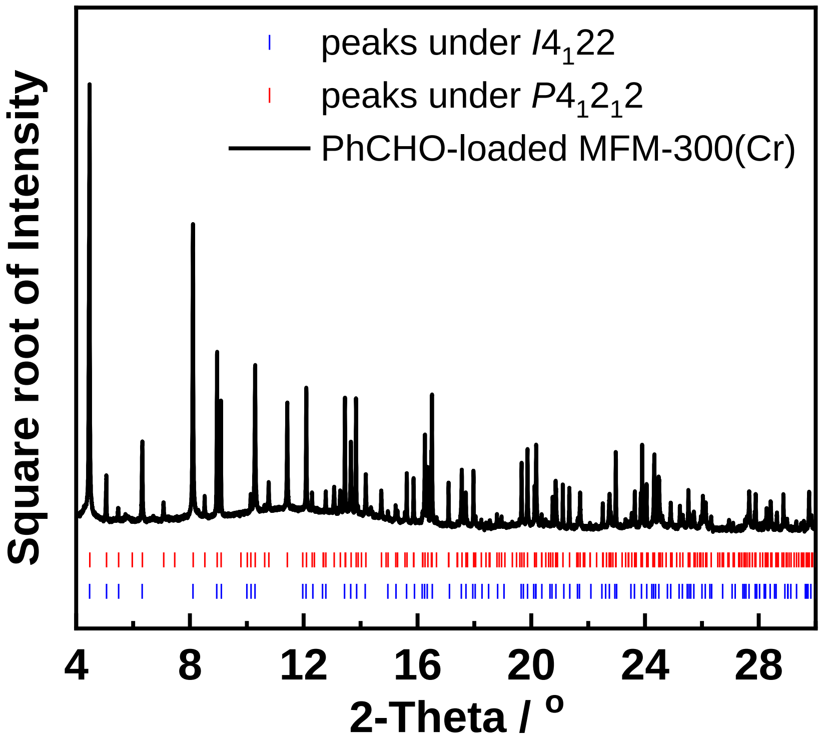

Supplementary Fig. 14. Observed SPXRD pattern for PhCHO-loaded MFM-300(Cr). Tick marks at the bottom of the image show allowed Bragg reflection positions for space groups $P4_12_12$ and $I4_122$ (red and blue, respectively).

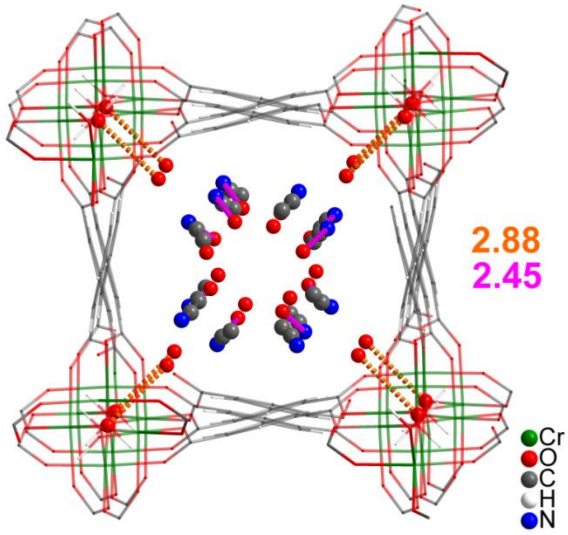

Supplementary Fig. 15. View of the crystal structure of CH_3CN-H_2O@MFM-300(Cr). This model was obtained from Rietveld refinements based on SPXRD data.

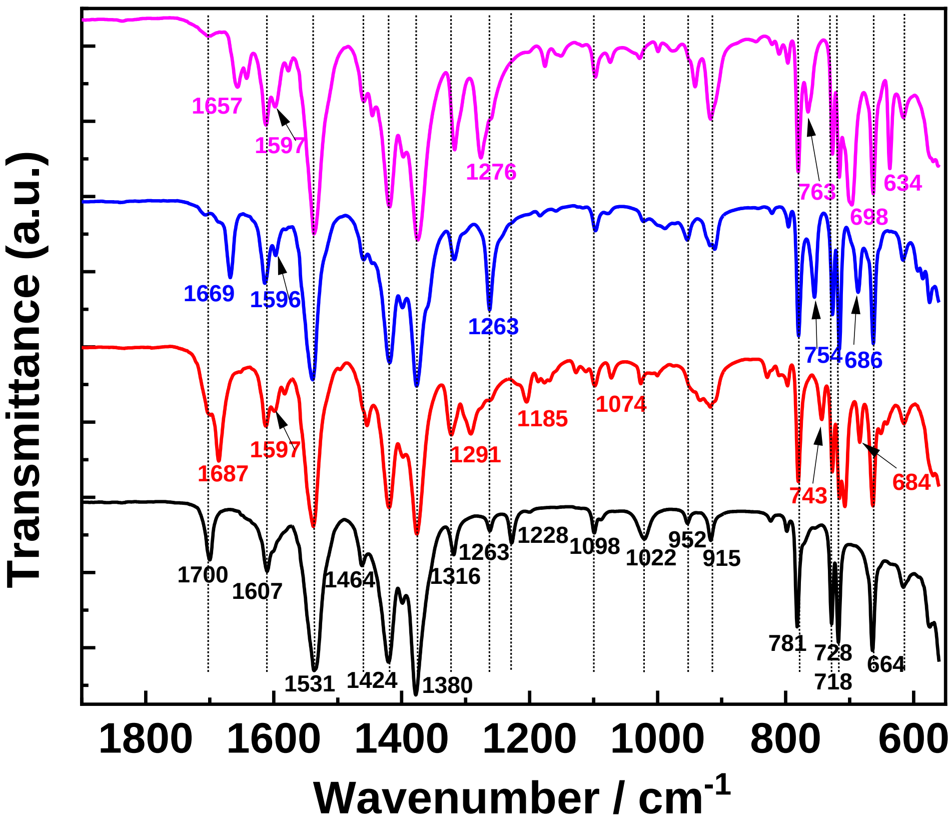

Supplementary Fig. 16. FT-IR spectra of as-synthesised MFM-300(Cr) (black), PhCHO-loaded MFM-300(Cr) (red), $PhCOCH_3$-loaded MFM-300(Cr) (blue) and Ph_2CO-loaded MFM-300(Cr) (pink) in the region of 1900-550 cm^{-1}.

The adsorption of substrates (PhCHO, $PhCOCH_3$ and Ph_2CO) in MFM-300(Cr) and the formation of host-guest binding interactions have also been confirmed by FTIR spectra (Supplementary Fig. 16). Compared with the bulk phase, the distinguishing peak at 1700 cm^{-1}, assigned to the C=O stretching vibration, is redshifted to 1687, 1669 and 1657 cm^{-1} for PhCHO@MFM-300(Cr), $PhCOCH_3$@MFM-300(Cr) and Ph_2CO@MFM-300(Cr), respectively.[2,3] The IR bands at 1263 and 1276 cm^{-1} are assigned to the stretching vibration of C-C-C groups of the adsorbed $PhCOCH_3$ and Ph_2CO molecules, respectively.[2, 3] Four IR bands at 1531, 1463, 1424 and 1380 of bare MFM-300(Cr) are assigned to the skeletal vibration of phenyl rings of the organic ligands, and the peaks at 781-664 are assigned to the bending modes of the aromatic C-H groups.[4] Upon adsorption of the substrates, small changes are observed for the vibrational modes of the MOF host, consistent with the formation of host-guest binding interactions, as confirmed by the crystallographic analysis.

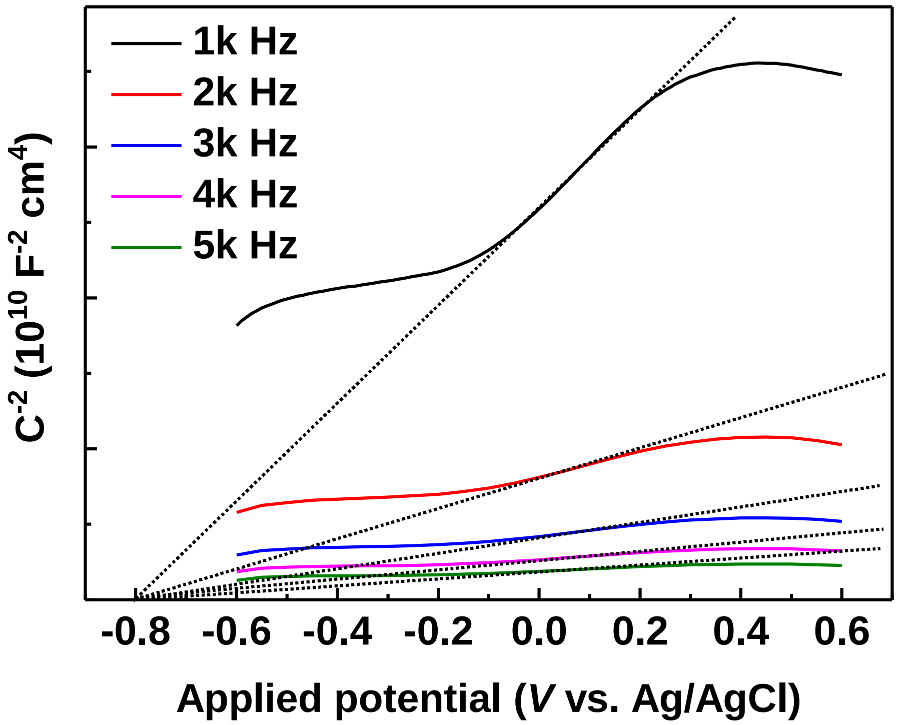

Supplementary Fig. 17. Mott-Schottky plots for MFM-300(Cr) at frequencies of 1k, 2k, 3k, 4k and 5k Hz.

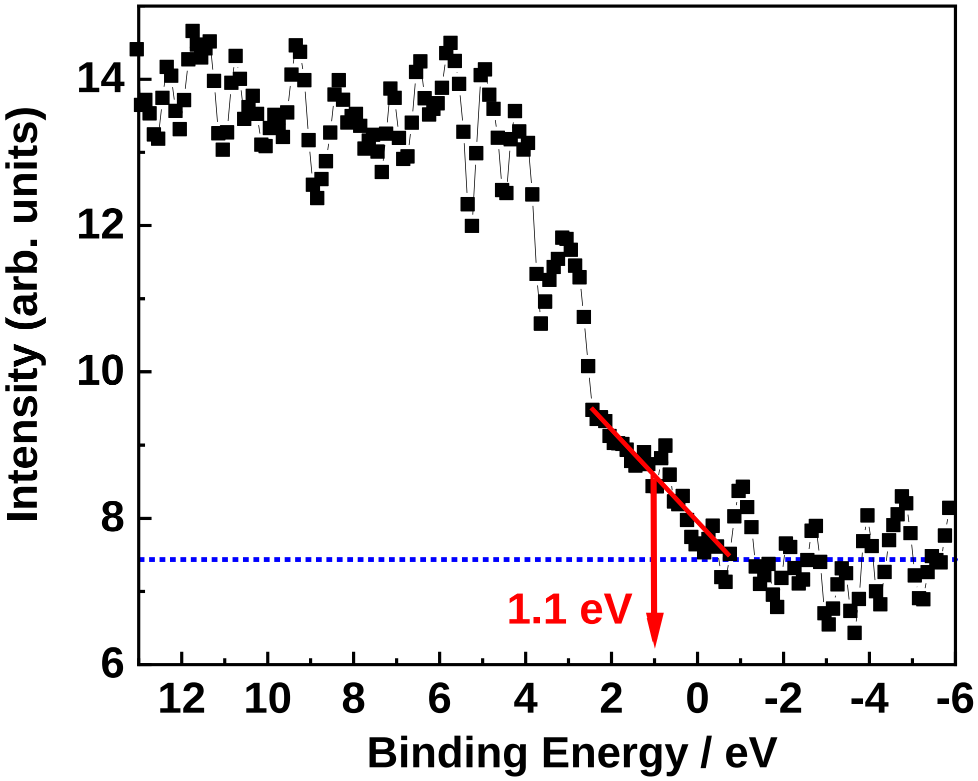

Supplementary Fig. 18. Valence band-XPS spectrum of MFM-300(Cr).

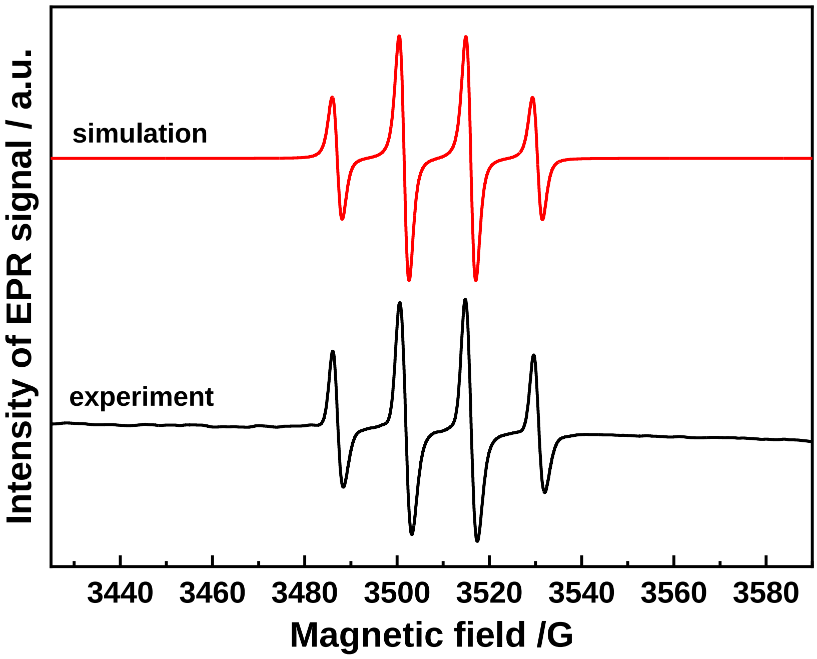

Supplementary Fig. 19. *In situ* EPR spectra of photoreductive coupling reaction. X-band EPR spectra of *in situ* photocatalytic reaction with 50% H_2O (CH_3CN/H_2O = 1.5 mL/1.5 mL), experimental spectrum (black), simulated spectrum (red). Conditions: acetophenone (0.050 mmol), Na_2SO_3 (0.25 M), MFM-300(Cr) (10%, 0.005 mmol), DMPO (176.7 mmol/L), 350-780 nm irradiation for 10 minutes.

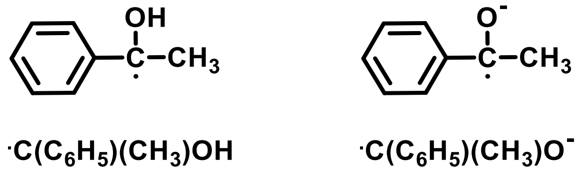

Supplementary Fig. 20. Carbon-centred radicals produced in the photo-catalysis reaction.

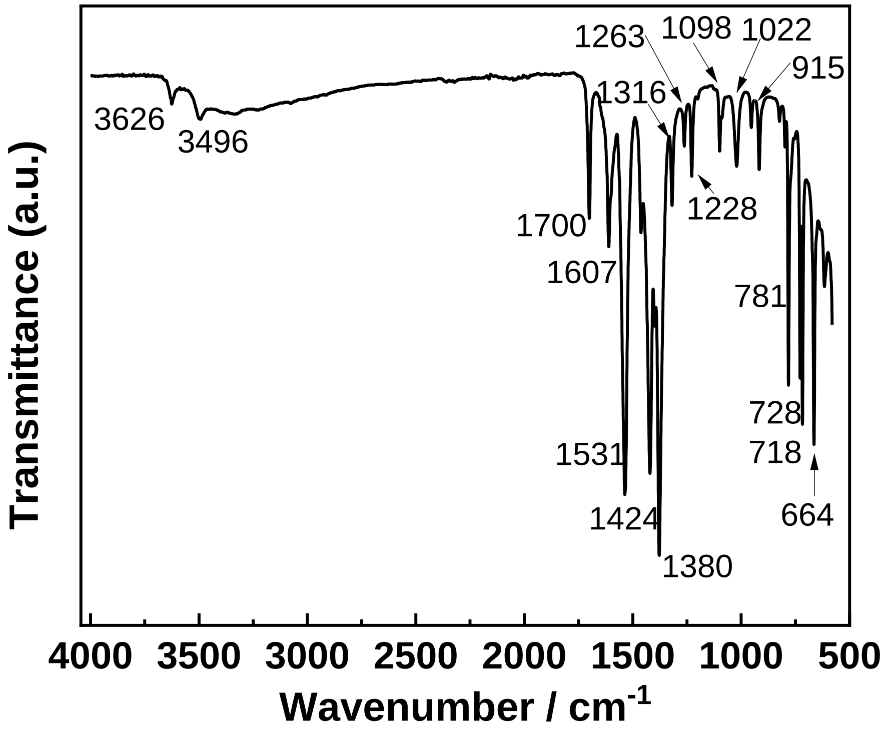

Supplementary Fig. 21. FT-IR spectrum of as-synthesised MFM-300(Cr).

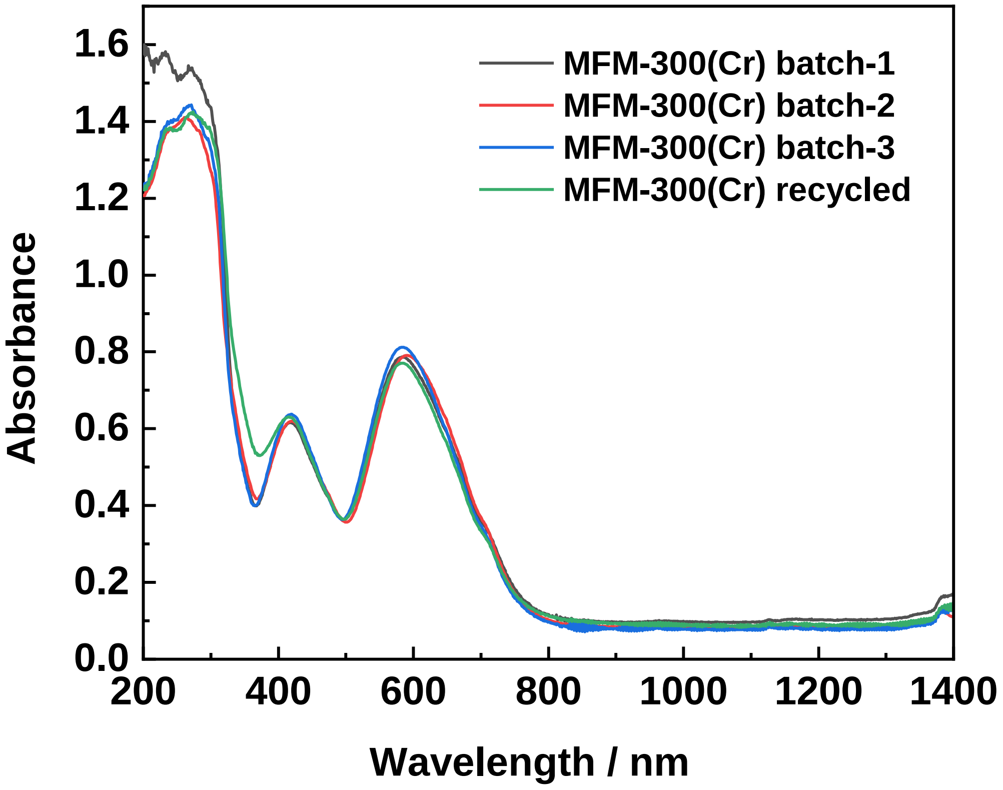

Supplementary Fig. 22. UV/Vis diffuse reflectance spectra of MFM-300(Cr) synthesised from three different batches and recycled after photocatalysis. The band gaps of the three batches and the recycled sample are 1.75, 1.75, 1.76 and 1.76 eV, respectively.

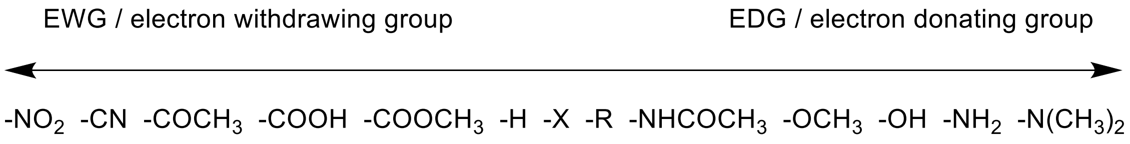

Supplementary Fig. 23. The electron donating/withdrawing ability of a variety of functional groups.

^{1}H NMR and ^{13}C NMR spectroscopy

1,2-Diphenylethane-1,2-diol (2a)

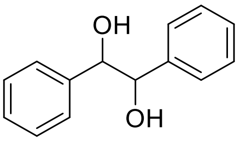

2a was prepared according to the general procedure using benzaldehyde (0.0545 g, 0.514 mmol) as the starting material. 0.0550 g **2a** was obtained in >99 % yield after 48 h. *meso* : *dl* = 63% : 37%. ^{1}H NMR of (*meso*)- and (*dl*)- **2a** (500 MHz, DMSO-d_6) δ 7.27-7.05 (m, 10H), 5.36 (*dl*) and 5.21 (*meso*) (s, 2H), 4.57 (*dl*) and 4.57 (*meso*) (s, 2H); ^{13}C NMR of (*meso*)- and (*dl*)- **2a** (125 MHz, DMSO-d_6) δ 143.2, 142.3, 127.3, 127.2 (× 2), 127.1, 126.7, 126.6, 77.6, 77.0. Spectral data for **2a** are in accordance with previous reports.[5-7] Benzaldehyde was used as a model substate in this study. The final product of **2a** was purified by column chromatography and the ^{1}H NMR and ^{13}C NMR spectra of purified **2a** are given below.

1,2-di-*p*-Tolylethane-1,2-diol (2b)

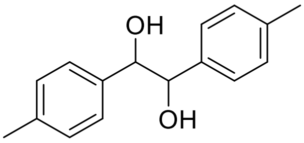

2b was prepared according to the general procedure using *p*-tolualdehyde (0.0620 g, 0.516 mmol) as starting material. 0.0570 g **2b** was obtained in 91 % yield after 12 h. *meso* : *dl* = 42% : 58%. ^{1}H NMR of (*meso*)- and (*dl*)- **2b** (500 MHz, $CDCl_3$) δ 7.09-7.03 (*meso*) and 7.00-6.93 (*dl*) (m, 8H), 4.70 (*meso*) and 4.55 (*dl*) (s, 2H), 2.29 (*meso*) and 2.25 (*dl*) (s, 6H); ^{13}C NMR of (*meso*)- and (*dl*)- **2b** (125 MHz, $CDCl_3$) δ 137.2 (× 3), 137.1, 128.6, 128.5, 127.0, 126.9, 78.6, 77.7, 21.0 (× 2). Spectral data for **2b** are in accordance with previous reports.[7, 8]

1,2-di-o-Tolylethane-1,2-diol (2c)

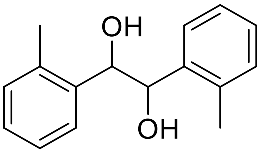

2c was prepared according to the general procedure using 2-Methyl benzaldehyde (0.0642 g, 0.535 mmol) as starting material. 0.0538 g **2c** was obtained in 83 % yield after 12 h. *meso* : *dl* = 42% : 58%. ^{1}H NMR of (*meso*)- and (*dl*)- **2c** (500 MHz, $CDCl_3$) δ 7.59 (d, *J* = 6.7 Hz), 7.55 (*d*, *J* = 7.0 Hz), 7.38 (d, *J* = 5.9 Hz), 7.31-7.00 (m) and 6.90 (d, *J* = 7.2 Hz) (*meso* and *dl*, 8H), 5.15 (*meso*) and 4.91 (*dl*) (s, 2H), 2.12 (*meso*) and 1.65 (*dl*) (s, 6H); ^{13}C NMR of (*meso*)- and (*dl*)- **2c** (125 MHz, $CDCl_3$) δ 138.2, 138.1, 136.2, 136.0, 130.2, 130.1, 127.8 (× 2), 127.2, 126.7, 126.1, 126.0, 74.7, 73.4, 19.2, 18.8. Spectral data for **2c** are in accordance with previous reports.[5]

1,2-di-m-Tolylethane-1,2-diol (2d)

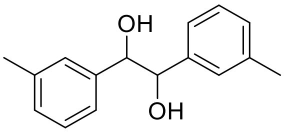

2d was prepared according to the general procedure using 3-Methyl benzaldehyde (0.0630 g, 0.525 mmol) as starting material. 0.0629 g **2d** was obtained in 99 % yield after 24 h. *meso* : *dl* = 58% : 42%. ^{1}H NMR of (*meso*)- and (*dl*)- **2d** (500 MHz, DMSO-d_6) δ 7.17-6.88 (m, 8H), 4.53 (*meso*) and 4.51 (*dl*) (s, 2H), 2.28 (*dl*) and 2.22 (*meso*) (s, 6H); ^{13}C NMR of (*meso*)- and (*dl*)- **2d** (125 MHz, DMSO-d_6) δ 143.5, 142.4, 136.2 (× 2), 128.1, 127.8, 127.3, 127.2 (× 3), 124.6, 124.3, 77.7, 77.1, 21.1, 21.0. Spectral data for **2d** are in accordance with previous reports.[9]

1,2-Bis(3,5-dimethylphenyl)ethane-1,2-diol (2e)

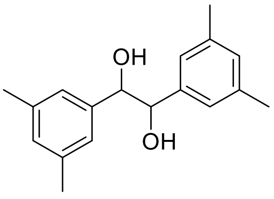

2e was prepared according to the general procedure using 3, 5-Dimethylbenzaldehyde (0.0675 g, 0.503 mmol) as starting material. 0.0645 g **2e** was obtained in 95 % yield after 12 h. *meso* : *dl* = 39% : 61%. ^{1}H NMR of (*meso*)- and (*dl*)- **2e** (500 MHz, $CDCl_3$) δ 6.83 (*meso*) and 6.77 (*dl*) (s, 2H), 6.81 (*meso*) and 6.67 (*dl*) (s, 4H), 4.54 (*meso*) and 4.47 (*dl*) (s, 2H), 2.20 (*meso*) and 2.15 (*dl*) (s, 12H); ^{13}C NMR of (*meso*)- and (*dl*)- **2e** (125 MHz, $CDCl_3$) δ 140.2 (× 2), 138.0, 137.6, 130.0, 129.4, 125.1, 124.6, 78.5, 78.4, 21.4 (× 2), 21.3 (× 2). Spectral data for **2e** are in accordance with previous reports.[10]

1,2-Bis(4-methoxyphenyl)ethane-1,2-diol (2f)

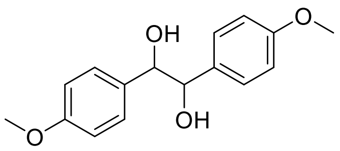

2f was prepared according to the general procedure using 4-Methoxybenzaldehyde (0.0690 g, 0.507 mmol) as starting material. 0.0690 g **2f** was obtained in 99 % yield after 24 h. *meso* : *dl* = 58% : 42%. ^{1}H NMR of (*meso*)- and (*dl*)- **2f** (500 MHz, DMSO-d_6) δ 7.17 (*dl*) and 7.01 (*meso*) (m, 4H), 6.82 (*dl*) and 6.74 (*meso*) (m, 4H), 5.22 (*meso*) and 5.04 (*dl*) (s, 2H), 4.53 (*dl*) and 4.50 (*meso*) (s, 2H), 3.72 (*dl*) and 3.69 (*meso*) (s, 6H); ^{13}C NMR of (*meso*)- and (*dl*)- **2f** (125 MHz, DMSO-d_6) δ 158.2 (× 2), 135.3, 134.3, 128.5, 128.4, 112.8, 112.7, 77.6, 76.7, 54.9 (× 2). Spectral data for **2f** are in accordance with previous reports.[5-7, 11]

1,2-Bis(4-ethylphenyl)ethane-1,2-diol (2g)

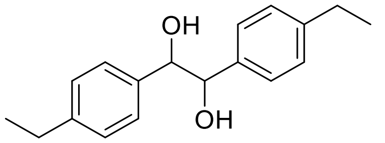

2g was prepared according to the general procedure using 4-Ethylbenzaldehyde (0.0675 g, 0.503 mmol) as starting material. 0.0572 g **2g** was obtained in 84 % yield after 24 h. *meso* : *dl* = 60% : 40%. ^{1}H NMR of (*meso*)- and (*dl*)- **2g** (500 MHz, DMSO-d_6) δ 7.21 (m, 4H, *dl*), 7.10 (m, 4H, *dl*), 7.07-7.00 (m, 8H, *meso*), 5.22 (*meso*) and 5.07 (*dl*) (s, 2H), 4.55 (*meso*) and 4.53 (*dl*) (s, 2H), 2.58 (*dl*) and 2.54 (*meso*) (q, J = 7.6 Hz,, 4H), 1.18 (*dl*) and 1.14 (*meso*) (t, J = 7.6 Hz, 6H); ^{13}C NMR of (*meso*)- and (*dl*)- **2g** (125 MHz, DMSO-d_6) δ 142.1, 139.8, 129.7, 128.6, 127.4, 127.2, 126.7 (× 2), 77.5, 77.0, 28.0, 27.9, 15.7, 15.6. Spectral data for **2g** are in accordance with previous reports.[12]

1,2-Bis(4-fluorophenyl)ethane-1,2-diol (2h)

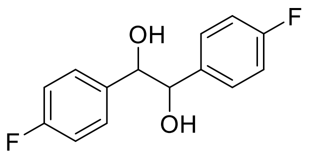

2h was prepared according to the general procedure using 4-Fluorobenzaldehyde (0.0653 g, 0.526 mmol) as starting material. 0.0553 g **2h** was obtained in 84 % yield after 24 h. *meso* : *dl* = 37% : 63%. ^{1}H NMR of (*meso*)- and (*dl*)- **2h** (500 MHz, $CDCl_3$) δ 7.04-6.98 (*meso*) and 6.96-6.90 (*dl*) (m, 4H), 6.86-6.81 (*meso*) and 6.81-6.76 (*dl*) (m, 4H), 4.71 (*meso*) and 4.47 (*dl*) (s, 2H), 3.75 (*dl*) and 3.22 (*meso*) (br, 2H); ^{13}C NMR of (*meso*)- and (*dl*)- **2h** (125 MHz, $CDCl_3$) δ 166.4 (d, $J_{C\text{-}F}$ = 256.4 Hz), 162.0 (d, $J_{C\text{-}F}$ = 245.5 Hz), 135.8 (d, $J_{C\text{-}F}$ = 3.1 Hz), 135.6 (d, $J_{C\text{-}F}$ = 3.0 Hz), 128.6 (d, $J_{C\text{-}F}$ = 8.1 Hz), 128.6 (d, $J_{C\text{-}F}$ = 8.0 Hz), 114.6 (d, $J_{C\text{-}F}$ = 21.3 Hz), 114.5 (d, $J_{C\text{-}F}$ = 21.3 Hz), 78.3, 76.8. Spectral data for **2h** are in accordance with previous reports.[5, 6, 9, 13]

1,2-Bis(4-(*tert*-butyl)phenyl)ethane-1,2-diol (2i)

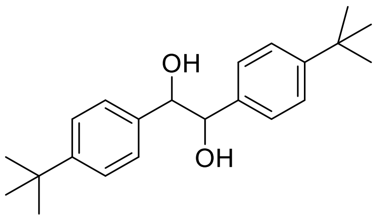

2i was prepared according to the general procedure using 4-(*tert*-butyl) benzaldehyde (0.1091 g, 0.673 mmol) as the starting material. 0.0807 g **2i** was obtained in >99 % yield after 24 h. *meso* : *dl* = 41% : 59%. ^{1}H NMR of (*meso*)- and (*dl*)- **2i** (500 MHz, DMSO-d_6) δ 7.34-7.26 (m, 8H, *meso*), 7.24 (d, J = 8.0 Hz, 4H, *dl*) and 7.15(d, J = 8.0 Hz, 4H, *dl*), 4.58 (*dl*) and 4.47 (*meso*) (s, 2H), 1.30 (*meso*) and 1.26 (*dl*) (s, 18H); ^{13}C NMR of (*meso*)- and (*dl*)- **2i** (125 MHz, DMSO-d_6) δ 149.0 (× 2), 141.4, 140.1, 127.3, 127.0, 124.2 (× 2), 77.2, 77.0, 34.3, 34.2, 31.4, 31.3. Spectral data for **2i** are in accordance with previous reports.[5, 14]

1,2-Di(4-biphenyl)-1,2-ethanediol (2j)

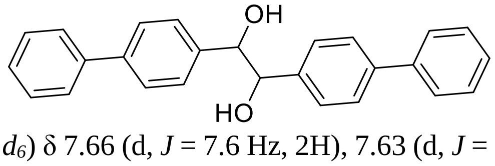

2j was prepared according to the general procedure using 4-iobobenzaldehyde (0.0911 g, 0.500 mmol) as the starting material. 0.0888 g **2j** was obtained in 97 % yield after 24 h. *meso* : *dl* = 50% : 50%. ^{1}H NMR of (*meso*)- and (*dl*)- **2j** (500 MHz, DMSO-d_6) δ 7.66 (d, J = 7.6 Hz, 2H), 7.63 (d, J = 7.6 Hz, 2H), 7.59 (d, J = 8.0 Hz, 2H), 7.52 (d, J = 8.0 Hz, 2H), 7.48-7.40 (m, 6H), 7.38-7.32 (m, 2H), 7.30 (d, J = 8.2 Hz, 2H), 5.47 (*meso*) and 5.36 (*dl*) (s, 2H), 4.75 (*meso*) and 4.70 (*dl*) (s, 2H); ^{13}C NMR of (*meso*)- and (*dl*)- **2j** (125 MHz, DMSO-d_6) δ 143.0, 141.9, 140.4, 140.2, 138.7 (× 2), 129.0 (× 2), 128.2, 128.0, 127.3 (× 2), 126.7, 126.6, 125.8 (× 2), 77.4, 77.0. Spectral data for **2j** are in accordance with previous reports.[5]

1,2-di(Naphthalen-1-yl)ethane-1,2-diol (2k)

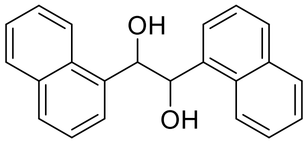

2k was prepared according to the general procedure using 1-Naphthaldehyde (0.0785 g, 0.503 mmol) as starting material. 0.0642 g **2k** was obtained in 81 % yield after 12 h. *meso* : *dl* = 54% : 46%. ^{1}H NMR of (*meso*)- and (*dl*)- **2k** (500 MHz, $CDCl_3$) δ 7.80-7.02 (m, *meso* and *dl*, 14H), 5.80 (*meso*) and 5.54 (*dl*) (s, 2H), 3.76 (*dl*) and 3.09 (*meso*) (br, 2H); ^{13}C NMR of (*meso*)- and (*dl*)- **2k** (125 MHz, $CDCl_3$) δ 136.2, 135.9, 133.5, 133.3, 131.4, 130.8, 128.6, 128.5, 128.3, 128.2, 125.8, 125.5, 125.3, 125.2, 125.1, 125.0, 124.9, 124.8, 123.0 (× 2), 74.4, 73.8. Spectral data for **2k** are in accordance with previous reports.[13]

1,2-di(Naphthalen-2-yl)ethane-1,2-diol (2l)

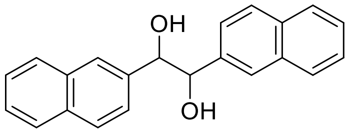

2l was prepared according to the general procedure using 2-Naphthaldehyde (0.0790 g, 0.506 mmol) as starting material. 0.0669 g **2l** was obtained in 84 % yield after 12 h with by-product 11.3% (conversion = 95.3%). *meso* : *dl* = 95% : 5%. ^{1}H NMR of (*meso*)-**2l** (500 MHz, DMSO-d_6) δ 7.81-7.75 (m, 2H), 7.74-7.69 (m, 2H), 7.67(s, 1H), 7.67-7.64 (m, 3H), 7.42-7.37 (m, 4H), 7.32-7.27 (m, 2H), 5.54 (br, 2H), 4.89 (s, 2H); ^{13}C NMR of (*meso*)-**2l** (125 MHz, DMSO-d_6) δ 140.2, 132.6, 132.3, 127.7, 127.4, 126.7, 125.8 (× 2), 125.7, 125.5, 77.6. Spectral data for **2l** are in accordance with previous reports.[9, 13]

2,3-Diphenyl-butane-2,3-diol (3a)

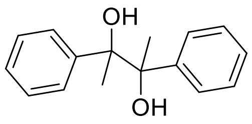

3a was prepared according to the general procedure using acetophenone (0.065 g, 0.541 mmol) as starting material. 0.0644 g **3a** was obtained in 98 % yield after 24 h. *meso* : *dl* = 39% : 61%. ^{1}H NMR of (*meso*)- and (*dl*)-**3a** (500 MHz, $CDCl_3$) δ 7.26-7.17 (m, 10H), 2.63 (*dl*) and 2.32 (*meso*) (s, 2H), 1.57 (*meso*) and 1.49 (*dl*) (s, 6H); ^{13}C NMR of (*meso*)- and (*dl*)-**3a** (125 MHz, $CDCl_3$) δ 143.8, 143.5, 127.4, 127.3, 127.2, 127.1, 127.0 (× 2), 78.9, 78.7, 25.2, 25.0. Spectral data for **3a** are in accordance with previous reports.[5, 6, 9, 11] Acetophenone was used as a model substate in this study. The final product of **3a** was purified by column chromatography and the ^{1}H NMR and ^{13}C NMR spectra of purified **3a** are given below.

2,3-di-p-Tolyl-butane-2,3-diol (3b)

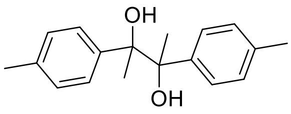

3b was prepared according to the general procedure using 4'-Methylacetophenone (0.0680 g, 0.507 mmol) as starting material. 0.0667 g **3b** was obtained in 97 % yield after 24 h. *meso* : *dl* = 53% : 47%. ^{1}H NMR of (*meso*)- and (*dl*)-**3b** (500 MHz, DMSO-d_6) δ 7.28 (d, J = 8.2 Hz, 4H, *dl*), 6.99 (d, J = 8.0 Hz, 4H, *dl*), 6.97 (d, J = 8.2 Hz, 4H, *meso*), 6.90 (d, J = 8.0 Hz, 4H, *meso*), 4.88 (*meso*) and 4.75 (*dl*) (br, 2H), 2.24 (*dl*) and 2.20 (*meso*) (s, 6H), 1.40 (*meso*) and 1.29 (*dl*) (s, 6H); ^{13}C NMR of (*meso*)- and (*dl*)-**3b** (125 MHz, DMSO-d_6) δ 144.0, 143.0, 135.0, 134.9, 127.8, 127.6, 127.4, 127.2, 77.8, 77.4, 25.4, 25.0, 21.1, 20.9. Spectral data for **3b** are in accordance with previous reports.[6, 11]

2,3-di-*m*-Tolyl-butane-2,3-diol (3c)

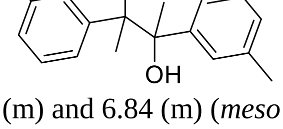

3c was prepared according to the general procedure using 3'-Methylacetophenone (0.0690 g, 0.515 mmol) as starting material. 0.0681 g **3c** was obtained in 98 % yield after 42 h. *meso* : *dl* = 61% : 39%. ^{1}H NMR of (*meso*)- and (*dl*)-**3c** (500 MHz, DMSO-d_6) δ 7.26-7.21 (m), 7.11 (t, J = 7.6 Hz), 7.02 (t, J = 7.6 Hz), 6.98 (m), 6.94 (m), 6.90 (m) and 6.84 (m) (*meso* and *dl*, 8H), 4.87 (*meso*) and 4.76 (*dl*) (s, 2H), 2.27 (*dl*) and 2.18 (*meso*) (s, 6H), 1.42 (*meso*) and 1.29 (*dl*) (s, 6H); ^{13}C NMR of (*meso*)- and (*dl*)-**3c** (125 MHz, DMSO-d_6) δ 146.8, 145.6, 134.9, 134.7, 128.4, 128.3, 126.4, 126.3 (× 2), 126.0, 124.8, 124.6, 77.5, 77.0, 25.2, 24.6, 21.4, 21.2. High resolution MS (ESI$^-$): Calcd. for $C_{18}H_{22}O_2Cl$ $(M+Cl)^-$ 305.1308; found: 305.1320.

2,3-bis(3-methoxyphenyl)butan-2,3-diol (3d)

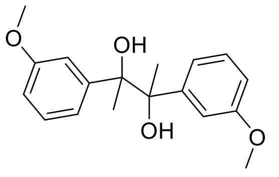

3d was prepared according to the general procedure using 3'- Methoxyacetophenone (0.0945 g, 0.630 mmol) as starting material. 0.0856 g **3d** was obtained in 90 % yield after 24 h. *meso* : *dl* = 62% : 38%. ^{1}H NMR of (*meso*)- and (*dl*)-**3d** (500 MHz, $CDCl_3$) δ 7.17-6.55 (*dl* and *meso*) (m, 8H), 4.98 (*meso*) and 4.88 (*dl*) (s, 2H), 3.68 (*dl*) and 3.56 (*meso*) (s, 6H), 1.48 (*meso*) and 1.30 (*dl*) (s, 6H); ^{13}C NMR of (*meso*)- and (*dl*)-**3d** (125 MHz, $CDCl_3$) δ 158.0, 157.7, 148.5, 147.8, 127.3, 127.0, 120.2, 120.0, 113.9, 113.4, 111.3, 111.1, 77.4, 77.1, 54.8, 54.6, 25.1, 24.7. Spectral data for **3d** are in accordance with previous reports.[15]

2,3-Bis(4-methoxyphenyl)butane-2,3-diol (3e)

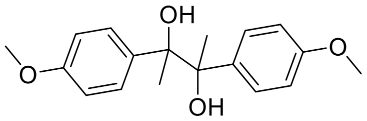

3e was prepared according to the general procedure using 4'-Methoxyacetophenone (0.0760 g, 0.506 mmol) as starting material. 0.0483 g **3e** was obtained in 63 % yield after 12 h. *meso* : *dl* = 41% : 59%. ^{1}H NMR of (*meso*)- and (*dl*)- **3e** (500 MHz, $CDCl_3$) δ 7.08-7.02 (*meso* and *dl*) (m, 4H), 6.72-6.66 (*meso* and *dl*) (m, 4H), 3.74 (*dl*) and 3.72 (*meso*) (s, 6H), 1.49 (*meso*) and 1.38 (*dl*) (s, 6H); ^{13}C NMR of (*meso*)- and (*dl*)- **3e** (125 MHz, $CDCl_3$) δ 163.1, 157.4, 130.5, 129.9, 128.6, 128.5, 111.7, 111.5, 77.3, 76.9, 54.9, 54.8, 25.2, 24.7. Spectral data for **3e** are in accordance with previous reports.[6, 11]

2,3-Bis(4-acetylamino-phenyl)butane-2,3-diol (3f)

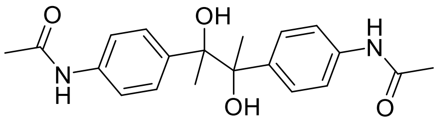

3f was prepared according to the general procedure using 4'-Acetamidoacetophenone (0.0890 g, 0.503 mmol) as starting material. 0.0644 g **3f** was obtained in 72 % yield after 24 h. *meso* : *dl* = 60% : 40%. ^{1}H NMR of (*meso*)- and (*dl*)- **3f** (500 MHz, DMSO-d_6) δ 9.82 (*dl*) and 9.78 (*meso*) (s, 2H), 7.56-6.90 (m, 8H), 4.86 (*meso*) and 4.76 (*dl*) (s, 2H), 2.03 (*dl*) and 2.01 (*meso*) (s, 6H), 1.44 (*meso*) and 1.29 (*dl*) (s, 6H); ^{13}C NMR of (*meso*)- and (*dl*)- **3f** (125 MHz, DMSO-d_6) δ 171.7, 168.1, 141.4, 140.7, 137.1 (× 2), 127.8, 127.6, 117.2, 116.9, 77.3, 77.0, 25.1, 24.7, 24.0, 22.5. High resolution MS (ESI$^+$): Calcd. for $C_{20}H_{24}O_4N_2Na$ $(M+Na)^+$ 379.1634; found: 379.1628.

2,3-Bis(4-ethylphenyl)butane-2,3-diol (3g)

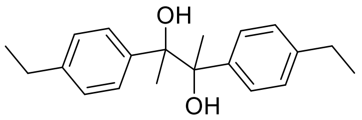

3g was prepared according to the general procedure using 4'-Ethylacetophenone (0.0750 g, 0.506 mmol) as starting material. 0.0740 g **3g** was obtained in 98 % yield after 24 h. *meso* : *dl* = 59% : 41%. ^{1}H NMR of (*meso*)- and (*dl*)- **3g** (500 MHz, DMSO-d_6) δ 7.38 (*dl*) (m, 4H), 7.06 (*dl*) (m, 4H), 7.03 (*meso*) (m, 4H) and 6.96 (*meso*) (m, 4H), 4.84 (*meso*) and 4.71 (*dl*) (s, 2H), 2.58 (q, *J* = 7.6 Hz, 4H, *dl*) and 2.54 (q, *J* = 7.5 Hz, 4H, *meso*), 1.42 (*meso*) and 1.28 (*dl*) (s, 6H), 1.18 (t, *J* = 7.6 Hz, 6H, *dl*) and 1.15 (t, *J* = 7.6 Hz, 6H, *meso*); ^{13}C NMR of (*meso*)- and (*dl*)- **3g** (125 MHz, DMSO-d_6) δ 144.2, 143.1, 141.1, 140.9, 127.6, 127.4, 125.8, 125.6, 77.4, 76.9, 27.8 (× 2), 25.2, 24.7, 15.7 (× 2). High resolution MS (ESI$^+$): Calcd. for $C_{20}H_{26}O_2Na$ $(M+Na)^+$ 321.1830; found: 321.1810.

2,3-Bis(4-fluorophenyl)butane-2,3-diol (3h)

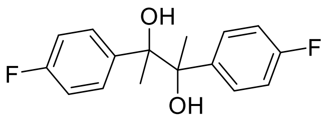

3h was prepared according to the general procedure using 4'-Fluoroacetophenone (0.0700 mg, 0.507 mmol) as starting material. 0.0475 g **3h** was obtained in 67 % yield after 12 h. *meso* : *dl* = 61% : 39%. ^{1}H NMR of (*meso*)- and (*dl*)- **3h** (500 MHz, $CDCl_3$) δ 7.31 (*dl*) (m, 4H), 7.06 (*meso*) (m, 4H), 7.00 (*dl*) (m, 4H), 6.84 (*meso*) (m, 4H), 5.07 (*meso*) and 4.96 (*dl*) (s, 2H), 1.54 (*meso*) and 1.29 (*dl*) (s, 6H); ^{13}C NMR of (*meso*)- and (*dl*)- **3h** (125 MHz, $CDCl_3$) δ 165.7 (d, J_{C-F} = 251.9 Hz), 161.5 (d, J_{C-F} = 241.7 Hz), 142.9 (d, J_{C-F} = 2.7 Hz), 142.7 (d, J_{C-F} = 2.7 Hz), 131.8 (d, J_{C-F} = 9.5 Hz), 129.9 (d, J_{C-F} = 7.8 Hz), 113.7 (d, J_{C-F} = 20.6 Hz), 113.3 (d, J_{C-F} = 18.9 Hz), 77.7, 77.5, 25.4, 25.1.[11]

2,3-Bis(4-(trifluoromethyl)phenyl)butane-2,3-diol (3i)

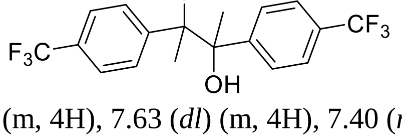

3i was prepared according to the general procedure using 4'-(Trifluoromethyl)acetophenone (0.0950 g, 0.505 mmol) as starting material. 0.0706 g **3i** was obtained in 74 % yield after 48 h. *meso* : *dl* = 59% : 41%. ^{1}H NMR of (*meso*)- and (*dl*)- **3i** (500 MHz, $CDCl_3$) δ 7.76 (*dl*) (m, 4H), 7.63 (*dl*) (m, 4H), 7.40 (*meso*) (m, 4H), 7.31 (*meso*) (m, 4H), 5.42 (*meso*) and 5.27 (*dl*) (s, 2H), 1.62

(*meso*) and 1.26 (*dl*) (s, 6H); ^{13}C NMR of (*meso*)- and (*dl*)- **3i** (125 MHz, $CDCl_3$) δ 147.7, 147.0, 129.1, 128.4, 127.7, 127.4, 123.4 (m), 122.9 (m), 77.0, 76.6, 24.8, 24.5. Spectral data for **3i** are in accordance with previous reports.[5, 16]

Dimethyl 4,4'-(2,3-dihydroxybutane-2,3-diyl)dibenzoate (3j)

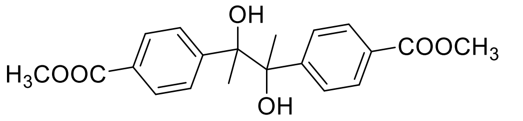

3j was prepared according to the general procedure using Methyl 4-acetylbenzoate (0.0900 g, 0.505 mmol) as starting material. 0.0453 g **3j** was obtained in 50 % yield after 48 h with by-product 38.2% (conversion = 88.2%). *meso* : *dl* = 55% : 45%. ^{1}H NMR of (*meso*)- and (*dl*)-**3j** (500 MHz, DMSO-d_6) δ 7.92 (m, 4H, *dl*), 7.64 (m, 4H, *meso*), 7.49 (m, 4H, *dl*), 7.22 (m, 4H, *meso*), 3.84 (*dl*) and 3.79 (*meso*) (s, 6H), 1.60 (*meso*) and 1.34 (*dl*) (s, 6H); ^{13}C NMR of (*meso*)- and (*dl*)- **3j** (125 MHz, DMSO-d_6) δ 152.9, 152.0, 129.2, 129.0, 127.5, 127.3 127.1, 127.0, 125.5 (× 2), 77.3, 67.7, 51.9, 51.8, 25.6, 24.5. Spectral data for **3j** are in accordance with previous reports.[17]

3,4-Diphenyl-hexane-3,4-diol (3k)

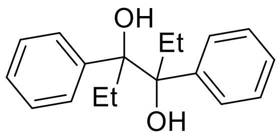

3k was prepared according to the general procedure using propiophenone (0.0675 g, 0.503 mmol) as starting material. 0.0647 g **3k** was obtained in 95 % yield after 48 h. *meso* : *dl* = 60% : 40%. ^{1}H NMR of (*meso*)- and (*dl*)-**3k** (500 MHz, DMSO-d_6) δ 7.38-6.94 (m, 10H), 4.56 (*meso*) and 4.47 (*dl*) (s, 2H), 2.24, 1.98, 1.88 and 1.37 (m, *meso* and *dl*, 4H), 0.53 (t, J = 7.3 Hz, 6H, *meso*) and 0.45 (t, J = 7.3 Hz, 6H, *dl*); ^{13}C NMR of (*meso*)- and (*dl*)-**3k** (125 MHz, DMSO-d_6) δ 143.3, 142.3, 128.2, 128.0, 126.4, 126.2, 125.6 (× 2), 81.1, 80.9, 27.7, 27.0, 8.0, 7.9. Spectral data for **3k** are in accordance with previous reports.[5]

2,3-Bis-(4-biphenylyl)-2,3-butanediol (3l)

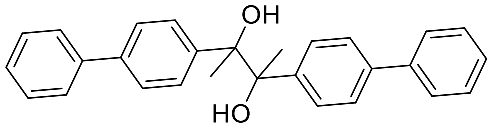

3l was prepared according to the general procedure using 4-acetylbiphenyl (0.0980 g, 0.499 mmol) as the starting material. 0.0759 g **3l** was obtained in 77 % yield after 24 h with 1-([1,1'-biphenyl]-4-yl)ethanol as by-product in 17% yield. *meso* : *dl* = 66% : 34%. ^{1}H NMR of (*meso*)- and (*dl*)- **3l** (500 MHz, DMSO-d_6) δ 7.71-7.20 (m, *meso* and *dl*, 18H), 5.12 (*meso*) and 5.02 (*dl*) (s, 2H), 1.57 (*meso*) and 1.38 (*dl*) (s, 6H); ^{13}C NMR of (*meso*)- and (*dl*)- **3l** (125 MHz, DMSO-d_6) δ 146.4, 145.5, 140.3, 140.1, 137.6, 137.4, 128.9 (× 2), 128.8 (× 2), 128.4, 128.1, 126.5, 124.8, 124.4, 77.4, 77.0, 25.2, 24.8. Spectral data for **3l** are in accordance with previous reports.[17]

2,3-Bis(p-cyanophenyl)-2,3-butanediol (3m)

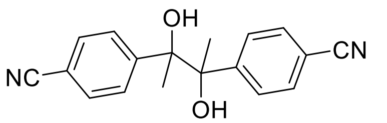

3m was prepared according to the general procedure using 4-Acetylbenzonitrile (0.0730 g, 0.503 mmol) as starting material. 0.0733 g **3m** was obtained in >99 % yield after 36 h. *meso* : *dl* = 76% : 24%. ^{1}H NMR of (*meso*)- and (*dl*)-**3m** (500 MHz, DMSO-d_6) δ 7.67-7.25 (m, 8H, *meso* and *dl*), 5.47 (*meso*) and 5.30 (*dl*) (s, 2H), 1.61 (*meso*) and 1.25 (*dl*) (s, 6H); ^{13}C NMR of (*meso*)- and (*dl*)-**3m** (125 MHz, DMSO-d_6) δ 152.4, 152.1, 130.6, 130.2, 128.7, 128.0, 119.2 (×2), 109.0, 108.5, 77.1, 76.8, 24.6, 24.3. Spectral data for **3m** are in accordance with previous reports.[18]

2,3-Di(4-methylmercaptophenyl)-2,3-butanediol (3n)

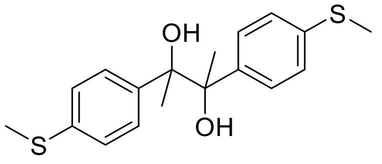

3n was prepared according to the general procedure using 1-(4-(methylthio)phenyl)ethan-1-one (0.0831 g, 0.500 mmol) as the starting material. 0.0758 g **3n** was obtained in 91 % yield after 24 h. *meso* : *dl* = 64% : 36%. ^{1}H NMR of (*meso*)- and (*dl*)- **3n** (500 MHz, DMSO-d_6) δ 7.33 (*dl*) and 7.07 (*dl*) (m, 4H), 7.03 (*meso*) and 6.97 (*meso*) (m, 4H), 4.82 (*meso*) and 4.71 (*dl*) (s, 2H), 2.42 (*dl*) and 2.39 (*meso*) (s, 6H), 1.41 (*meso*) and 1.28 (*dl*) (s, 6H); ^{13}C NMR of (*meso*)- and (*dl*)- **3n** (125 MHz, DMSO-d_6) δ 143.5, 142.5, 135.3, 135.1, 128.2, 128.0, 124.4, 124.1, 77.4, 76.9, 24.9, 24.5, 15.2, 15.1. Spectral data for **3n** are in accordance with previous reports.[17, 19]

Tetraphenylethane-1, 2-diol or benzopinacol (3o)

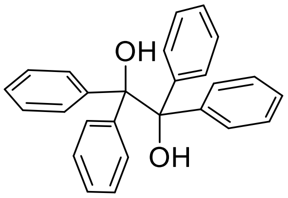

3o was prepared according to the general procedure using Benzophenone (0.0920 g, 0.505 mmol) as starting material. 0.0512 g **3o** was obtained in 55 % yield after 12 h. ^{1}H NMR (500 MHz, $CDCl_3$) δ 7.29-7.01 (m, 20H), 3.10 (br, 2H); ^{13}C NMR (125 MHz, $CDCl_3$) δ 143.9, 128.5, 127.5, 126.6, 83.0, 76.2. Spectral data for **3o** are in accordance with previous reports.[5, 7, 11, 13]

2,3-di-[2]Naphthyl-butane-2,3-diol (3p)

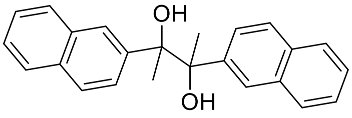

3p was prepared according to the general procedure using 2-Acetonaphthone (0.0860 g, 0.506 mmol) as starting material. 0.0799 g **3p** was obtained in 92 % yield after 12 h. *meso* : *dl* = 28% : 72%. ^{1}H NMR of (*meso*)- and (*dl*)-**3p** (500 MHz, $CDCl_3$) δ 7.90-7.68 (m), 7.56-7.32 (m, 14H, *meso* and *dl*), 3.53 (*dl*) and 3.09 (*meso*) (s, 2H), 1.77 (*meso*) and 1.67 (*dl*) (s, 6H); ^{13}C NMR of (*meso*)- and (*dl*)-**3p** (125 MHz, $CDCl_3$) δ 141.7, 141.2, 132.3, 132.2, 132.0, 131.9, 128.0, 127.0 (× 2), 126.2, 126.0, 125.9, 125.7 (× 2), 125.5 (× 2), 125.4, 78.8, 78.5, 24.9, 24.7. Spectral data for **3p** are in accordance with previous reports.[5]

1,1,2,2-Tetrakis(4-fluorophenyl)ethane -1,2-diol (3q)

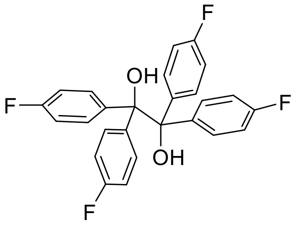

3q was prepared according to the general procedure using 4, 4'-Difluorobenzophenone (0.109 g, 0.500 mmol) as starting material. 0.0840 g **3q** was obtained in 77 % yield after 24 h. ^{1}H NMR (500 MHz, $CDCl_3$) δ 7.35-7.29 (m, 8H), 7.05-6.89 (m, 8H), 5.81 (s, 2H); ^{13}C NMR (125 MHz, $CDCl_3$) δ 162.4 (d, $J_{C\text{-}F}$ = 246.1 Hz), 139.6 (d, $J_{C\text{-}F}$ = 3.2 Hz), 128.3 (d, $J_{C\text{-}F}$ = 8.2 Hz), 115.5 (d, $J_{C\text{-}F}$ = 21.5 Hz), 75.1. Spectral data for **3q** are in accordance with previous reports.[5]

1,1,2,2-Tetra-p-tolylethane-1,2-diol (3r)

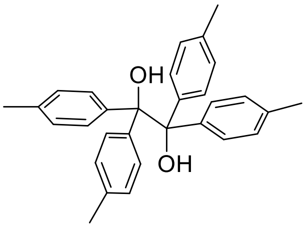

3r was prepared according to the general procedure using 4, 4'-Dimethylbenzophenone (0.105 g, 0.500 mmol) as starting material. 0.0845 g **3r** was obtained in 80 % yield after 24 h. ^{1}H NMR (500 MHz, DMSO-d_6) δ 7.19 (d, J = 8.2 Hz, 8H), 6.87 (d, J = 8.2 Hz, 8H), 5.56 (s, 2H), 2.20 (s, 12H); ^{13}C NMR (125 MHz, DMSO-d_6) δ 142.2, 132.7, 127.4, 125.0, 80.9, 18.8. Spectral data for **3r** are in accordance with previous reports.[7]

Supplementary Tables

Supplementary Table 1. Crystallographic data and details of bare and substrate-loaded MFM-300(Cr).

Sample	MeCN-water-loaded MFM-300(Cr)	PhCHO-loaded MFM-300(Cr)	$PhCOCH_3$-loaded MFM-300(Cr)	Ph_2CO-loaded MFM-300(Cr)
Crystal system	Tetragonal	Tetragonal	Tetragonal	Tetragonal
Space group	$I4_122$	$P4_12_12$	$I4_122$	$I4_122$
Chemical formula	$CrC_8O_5H_4\cdot$ 1.4 $CH_3CN\cdot$ 3.2 H_2O	$CrC_8O_5H_4\cdot$ 0.778 $C_7OH_6\cdot$0.923 H_2O	$CrC_8O_5H_4\cdot$ 0.778 $C_8OH_8\cdot$0.622 H_2O	$CrC_8O_5H_4\cdot$ 0.405 $C_{13}OH_{10}$
2θ range for refinement	4-50°	4-50°	4-50°	4-50°
Refinement methods	Rietveld Method	Rietveld Method	Rietveld Method	Rietveld Method
a (Å)	15.00438(5)	14.96702(2)	15.00160(2)	14.98943(3)
c (Å)	11.94560(4)	11.97932(2)	11.98765(2)	11.95527(5)
V (Å^3)	2689.33(2)	2683.51(1)	2697.80(1)	2686.15(2)
R_{wp} (%)	7.553	7.062	7.844	9.577
R_p(%)	5.675	5.509	6.096	7.000
R_{Bragg}(%)	3.605	2.577	3.770	4.622
R_{exp} (%)	2.321	2.403	2.301	2.105
Gof χ^2	3.253	2.939	3.409	4.550

Supplementary Table 2. Atomic parameters from the Rietveld refinement of MFM-300(Cr) in MeCN/H_2O.

Species	Atom	x	y	z	SOF	Beq (Å^2)	Wyckoff
MFM-300(Cr)	Cr	0.69264(5)	0.30736(5)	0.5	1	0.31(2)	8*e*
	O1	0.7575(3)	0.25	0.625	1	0.30(4)	8*f*
	O2	0.89898(17)	0.29037(17)	1	1	0.30(4)	16*g*
	O3	0.61981(18)	0.37598(19)	0.3900(2)	1	0.30(4)	16*g*
	C1	0.5895(3)	0.3650(3)	0.7056(3)	1	0.30(5)	16*g*
	C2	0.5415(3)	0.4317(3)	0.7652(4)	1	0.30(5)	16*g*
	C3	0.5	0.5	0.7005(4)	1	0.30(5)	8*c*
	C4	0.5433(3)	0.4276(3)	0.8823(3)	1	0.30(5)	16*g*
	C5	0.5	0.5	0.9371(4)	1	0.30(5)	8*c*
	H1	0.5	0.5	0.622	1	0.36(6)	8*c*
	H2	0.574	0.387	0.917	1	0.36(6)	16*g*
	H3	0.75	0.176	0.375	1	0.36(5)	8*f*
Water	OW1	0.3610(6)	0.2055(6)	0.0459(8)	0.743(11)	20.0(7)	16*g*
	OW2	-0.5531(4)	2.7530(7)	1.1654(7)	0.864(6)	15.9(4)	16*g*
MeCN	C_1	0.7038(5)	0.5934(4)	0.5201(9)	0.720(4)	18.3(4)	16*g*
	C_2	0.7(3)	0.62(14)	0.40(3)	0.720(4)	18.3(4)	16*g*
	N_1	0.7(2)	0.57(11)	0.61(3)	0.720(4)	18.3(4)	16*g*

Supplementary Table 3. Atomic parameters from the Rietveld refinement of PhCHO-loaded MFM-300(Cr).

Species	Atom	x	y	z	SOF	Beq (Å^2)	Wyckoff
MFM-300(Cr)	Cr1	0.55335(10)	0.06404(11)	0.2503(2)	1	0.38(2)	8*b*
	O2	0.5001(5)	-0.0033(2)	0.3754(10)	1	0.49(4)	8*b*
	O3	0.5278(4)	0.1525(3)	0.5088(6)	1	0.49(4)	8*b*
	O4	0.6155(4)	0.1424(4)	0.3562(5)	1	0.49(4)	8*b*
	O5	0.9488(4)	0.3551(3)	0.5047(6)	1	0.49(4)	8*b*
	O6	0.8592(4)	0.3828(4)	0.3612(5)	1	0.49(4)	8*b*
	C7	0.6005(6)	0.1641(6)	0.4569(8)	1	0.45(6)	8*b*
	C8	0.6648(6)	0.2229(6)	0.5168(9)	1	0.45(6)	8*b*
	C9	0.7314(7)	0.2633(7)	0.4526(4)	1	0.45(6)	8*b*
	C10	0.8049(6)	0.3043(5)	0.5100(9)	1	0.45(6)	8*b*
	C11	0.7991(7)	0.3049(6)	0.6279(7)	1	0.45(6)	8*b*
	C12	0.7278(7)	0.2730(7)	0.6890(4)	1	0.45(6)	8*b*
	C13	0.6621(6)	0.2311(6)	0.6328(7)	1	0.45(6)	8*b*
	C14	0.8786(6)	0.3524(6)	0.4489(8)	1	0.45(6)	8*b*
	H_O	0.499(6)	-0.073(3)	0.376(8)	1	0.59(5)	8*b*
Water	OW1	0.6471(7)	0.5267(7)	0.4666(11)	0.923(13)	20.0(8)	8*b*
PhCHO	C_1	0.4049(6)	0.4236(4)	-0.1756(6)	0.778(3)	10.0(3)	8*b*
	C_2	0.438(2)	0.3412(9)	-0.2133(7)	0.778(3)	10.0(3)	8*b*
	C_3	0.443(3)	0.3240(12)	-0.3285(7)	0.778(3)	10.0(3)	8*b*
	C_4	0.4158(18)	0.3887(9)	-0.4048(6)	0.778(3)	10.0(3)	8*b*
	C_5	0.3828(9)	0.4708(7)	-0.3666(6)	0.778(3)	10.0(3)	8*b*
	C_6	0.3774(14)	0.4884(7)	-0.2521(6)	0.778(3)	10.0(3)	8*b*
	C_7	0.467(3)	0.2725(14)	-0.1318(9)	0.778(3)	10.0(3)	8*b*
	O_1	0.497(5)	0.200(2)	-0.1563(11)	0.778(3)	10.0(3)	8*b*

Supplementary Table 4. Atomic parameters from the Rietveld refinement of $PhCOCH_3$-loaded MFM-300(Cr).

Species	Atom	x	y	z	SOF	Beq (Å^2)	Wyckoff
MFM-300(Cr)	Cr	0.69139	0.30861	0.5	1	0.66(2)	8*e*
	O1	0.7560(3)	0.25	0.625	1	0.49(4)	8*f*
	O2	0.89876(17)	0.28885(17)	1	1	0.49(4)	16*g*
	O3	0.6213(2)	0.3793(2)	0.3951(2)	1	0.49(4)	16*g*
	C1	0.5904(3)	0.3569(3)	0.6989(3)	1	0.47(5)	16*g*
	C2	0.5407(3)	0.4298(3)	0.7598(4)	1	0.47(5)	16*g*
	C3	0.5	0.5	0.7058(4)	1	0.47(5)	8*c*
	C4	0.5344(3)	0.4303(2)	0.8773(3)	1	0.47(5)	16*g*
	C5	0.5	0.5	0.9394(4)	1	0.47(5)	8*c*
	H1	0.5	0.5	0.622	1	0.56(6)	8*c*
	H2	0.574	0.387	0.917	1	0.56(6)	16*g*
	H3	0.75	0.176	0.375	1	0.59(5)	8*f*
Water	OW1	0.4252(19)	0.2328(18)	0.0069(18)	0.311(14)	20.0(16)	16*g*
$PhCOCH_3$	C_1	0.3257(5)	0.1771(7)	0.0500(8)	0.389(3)	10.0(3)	16*g*
	C_2	0.4093(13)	0.211(3)	0.0197(10)	0.389(3)	10.0(3)	16*g*
	C_3	0.4346(17)	0.213(4)	-0.0932(11)	0.389(3)	10.0(3)	16*g*
	C_4	0.3766(12)	0.181(2)	-0.1746(9)	0.389(3)	10.0(3)	16*g*
	C_5	0.2932(10)	0.1468(11)	-0.1438(8)	0.389(3)	10.0(3)	16*g*
	C_6	0.2676(10)	0.1449(17)	-0.0316(9)	0.389(3)	10.0(3)	16*g*
	C_7	0.471(2)	0.246(4)	0.1067(13)	0.389(3)	10.0(3)	16*g*
	C_8	0.4343(17)	0.239(3)	0.2264(11)	0.389(3)	10.0(3)	16*g*
	O_1	0.544(3)	0.276(6)	0.0886(17)	0.389(3)	10.0(3)	16*g*

Supplementary Table 5. Atomic parameters from the Rietveld refinement of Ph_2CO-loaded MFM-300(Cr).

Species	Atom	x	y	z	SOF	Beq (Å^2)	Wyckoff
MFM-300(Cr)	Cr	0.69141(7)	0.30859(7)	0.5	1	0.80(3)	8*e*
	O1	0.7570(3)	0.25	0.625	1	1.18(6)	8*f*
	O2	0.8979(2)	0.2882(2)	1	1	1.18(6)	16*g*
	O3	0.6191(2)	0.3757(3)	0.3940(3)	1	1.18(6)	16*g*
	C1	0.5886(4)	0.3625(3)	0.7065(4)	1	1.40(8)	16*g*
	C2	0.5393(4)	0.4318(3)	0.7563(6)	1	1.40(8)	16*g*
	C3	0.5	0.5	0.7021(6)	1	1.40(8)	8*c*
	C4	0.5372(4)	0.4329(3)	0.8766(4)	1	1.40(8)	16*g*
	C5	0.5	0.5	0.9391(5)	1	1.40(8)	8*c*
	H1	0.5	0.5	0.622	1	1.68(9)	8*c*
	H2	0.574	0.387	0.917	1	1.68(9)	16*g*
	H3	0.75	0.176	0.375	1	1.42(7)	8*f*
Ph_2CO	C_1	0.6097(8)	0.7990(7)	0.0318(12)	0.2027(16)	10.0(5)	16*g*
	C_2	0.5686(9)	0.7769(8)	0.1433(12)	0.2027(16)	10.0(5)	16*g*
	C_3	0.6191(12)	0.7581(12)	0.2394(13)	0.2027(16)	10.0(5)	16*g*
	C_4	0.4751(10)	0.7692(9)	0.1483(15)	0.2027(16)	10.0(5)	16*g*
	C_5	0.5769(14)	0.7330(14)	0.3388(13)	0.2027(16)	10.0(5)	16*g*
	C_6	0.4330(12)	0.7456(11)	0.2480(17)	0.2027(16)	10.0(5)	16*g*
	C_7	0.4839(14)	0.7274(13)	0.3435(15)	0.2027(16)	10.0(5)	16*g*
	C_8	0.6985(9)	0.8456(8)	0.0256(14)	0.2027(16)	10.0(5)	16*g*
	C_9	0.7285(11)	0.9059(11)	0.1074(16)	0.2027(16)	10.0(5)	16*g*
	C_10	0.7505(9)	0.8320(9)	-0.0708(15)	0.2027(16)	10.0(5)	16*g*
	C_11	0.8094(13)	0.9509(14)	0.093(2)	0.2027(16)	10.0(5)	16*g*
	C_12	0.8318(10)	0.8759(12)	-0.0838(19)	0.2027(16)	10.0(5)	16*g*
	C_13	0.8615(11)	0.9355(14)	-0.002(2)	0.2027(16)	10.0(5)	16*g*
	O_1	0.5707(9)	0.7789(9)	-0.0541(12)	0.2027(16)	10.0(5)	16*g*

Supplementary Table 6. Summary of simulation parameters of EPR spectra.

Samples	Adduct	g-factor	$A^{14}N$ / G	$A^{\beta}H$ / G	lw/mT
With 50% H_2O	DMPO*•OH	2.0057	14.5	14.5	0.18<br>0.18
With 13% H_2O	DMPO*•$C(C_6H_5)(CH_3)OH$	2.0055	14.8	22.0	0.1<br>0.41
	DMPO*•OH	2.0057	14.0	14.2	0.15<br>0.15
Without H_2O	DMPO*•$C(C_6H_5)(CH_3)OH$	2.0060	14.7	19.5	0.05<br>0.50

* lw is the homogeneous Lorentzian linewidth; *g* and *A* are the g and hyperfine constant parameters, respectively.

Supplementary References

1. Lin, X. *et al.*, High capacity hydrogen adsorption in Cu(II) tetracarboxylate framework materials: the role of pore size, ligand functionalization, and exposed metal sites. *J. Am. Chem. Soc.* **131**, 2159-2171 (2009).
2. Smith, B. C. The Carbonyl Group, Part I: Introduction. *Spectroscopy* **32**(9), 31-36 (2017).
3. Smith, B. C. The C=O Bond, Part II: Aldehydes. *Spectroscopy* **32**(11), 28-34 (2017).
4. Smith, B. C. Group wavenumbers and an introduction to the spectroscopy of benzene rings. *Spectroscopy* **31**(3), 34-37 (2016).
5. Nakajima, M., Fava, E., Loescher, S., Jiang, Z. & Rueping, M. Photoredox-catalyzed reductive coupling of aldehydes, ketones, and imines with visible light. *Angew. Chem. Int. Ed.* **54**, 8828-8832 (2015).
6. Uchiyama, M. *et al.* Development of a catalytic electron transfer system mediated by transition metal ate complexes: applicability and tunability of electron-releasing potential for organic transformations. *J. Am. Chem. Soc.* **126**, 8755-8759 (2004).
7. Guo, H. & Zhang, Y. A novel method for pinacolic coupling of aromatic aldehydes and ketones with a Sm–$ZnCl_2$ system. *J. Chem. Res.* **2000**, 284-286 (2000).
8. Zhou, Z.-Z., Liu, M., Lv, L. & Li, C.-J. Silver(I)-catalyzed widely applicable aerobic 1,2-diol oxidative cleavage. *Angew. Chem. Int. Ed.* **57**, 2616-2620 (2018).
9. Kim, Y. J., Kim, S. M., Hosono, H., Yang, J. W. & Kim, S. W. The scalable pinacol coupling reaction utilizing the inorganic electride $[Ca_2N]^+\cdot e^-$ as an electron donor. *Chem. Commun.* **50**, 4791-4794 (2014).
10. Hilgraf, R. & Pfaltz, A. Chiral bis(n-sulfonylamino)phosphine- and taddol-phosphite-oxazoline ligands: synthesis and application in asymmetric catalysis. *Adv. Synth. Catal.* **347**, 61-77 (2005).
11. Shi, H. *et al.* Lewis acid assisted electrophilic fluorine-catalyzed pinacol rearrangement of hydrobenzoin substrates: one-pot synthesis of (±)-latifine and (±)-cherylline. *J. Org. Chem.* **83**, 1312-1319 (2018).
12. Swift, J. A., Pal, R. & McBride, J. M. Using hydrogen-bonds and herringbone packing to design interfaces of 4,4‘-disubstituted meso-hydrobenzoin crystals. The importance of recognizing unfavorable packing motifs. *J. Am. Chem. Soc.* **120**, 96-104 (1998).
13. Gualandi, A. *et al*. Application of coumarin dyes for organic photoredox catalysis. *Chem. Commun.* **54**, 10044-10047 (2018).
14. Buchammagari, H. *et al.* Room temperature-stable electride as a synthetic organic reagent: application to pinacol coupling reaction in aqueous media. *Org. Lett.* **9**, 4287-4289 (2007)
15. Park, C.-H. & Givens, R. S. New photoactivated protecting groups. 6. p-hydroxyphenacyl: a phototrigger for chemical and biochemical probes. *J. Am. Chem. Soc.* **119**, 2453-2463 (1997).
16. Yoshimura, A., Saeki, T., Nomoto, A. & Ogawa, A. Pinacol couplings of a series of aldehydes and ketones with SmI_2/Sm/Me_3SiCl in DME. *Tetrahedron* **71**, 5347-5355 (2015).
17. Caron, A., Morin, É. & Collins, S. K. Bifunctional copper-based photocatalyst for reductive pinacol-type couplings. *ACS Catal.* **9**, 9458-9464 (2019).
18. Oku, A., Takahashi, H. & Asmus, S. M. Sequential two-electron transfer from tetraoxy-substituted cyclopropane to acceptors. *J. Am. Chem. Soc.* **122**, 7388-7389 (2000).
19. Charbonneau, L. F. & Smith, S. G. Kinetics of the reaction of butyllithium with 4-methylmercaptoacetophenone in benzene. *J. Org. Chem.* **41**, 808-812 (1976).
